# Advances in Mn-Based Electrode Materials for Aqueous Sodium-Ion Batteries

**DOI:** 10.1007/s40820-023-01162-x

**Published:** 2023-08-09

**Authors:** Changsheng Ding, Zhang Chen, Chuanxiang Cao, Yu Liu, Yanfeng Gao

**Affiliations:** 1 School of Materials Science and Engineering, Shanghai University, Shanghai, 200444 People’s Republic of China; 2 Shanghai Institute of Ceramics, Chinese Academy of Sciences, Shanghai, 200050 People’s Republic of China; 3 Key Laboratory of Comprehensive and Highly Efficient Utilization of Salt Lake Resources, Qinghai Institute of Salt Lakes, Chinese Academy of Sciences, Xining, 81000 People’s Republic of China

**Keywords:** Sodium-ion batteries, Aqueous electrolytes, Mn-based electrode materials, Electrochemical performance, Improvement methods

## Abstract

Aqueous sodium-ion batteries have attracted extensive attention for large-scale energy storage applications, due to abundant sodium resources, low cost, intrinsic safety of aqueous electrolytes and eco-friendliness. The electrochemical performance of aqueous sodium-ion batteries is affected by the properties of electrode materials and electrolytes. Among various electrode materials, Mn-based electrode materials have attracted tremendous attention because of the abundance of Mn, low cost, nontoxicity, eco-friendliness and interesting electrochemical performance. Aqueous electrolytes having narrow electrochemical window also affect the electrochemical performance of Mn-based electrode materials. In this review, we introduce systematically Mn-based electrode materials for aqueous sodium-ion batteries from cathode and anode materials and offer a comprehensive overview about their recent development. These Mn-based materials include oxides, Prussian blue analogues and polyanion compounds. We summarize and discuss the composition, crystal structure, morphology and electrochemical properties of Mn-based electrode materials. The improvement methods based on electrolyte optimization, element doping or substitution, optimization of morphology and carbon modification are highlighted. The perspectives of Mn-based electrode materials for future studies are also provided. We believe this review is important and helpful to explore and apply Mn-based electrode materials in aqueous sodium-ion batteries.

## Highlights

- Mn-based electrode materials, including oxides, Prussian blue analogues and polyanion compounds, are introduced systematically for aqueous sodium-ion batteries.
- The composition, crystal structure, morphology and electrochemical performance of Mn-based electrode materials are reviewed.
- The improvement methods of electrochemical performance, such as electrolyte optimization, element doping or substitution, morphology optimization and carbon modification, are discussed.

## Graphical abstract

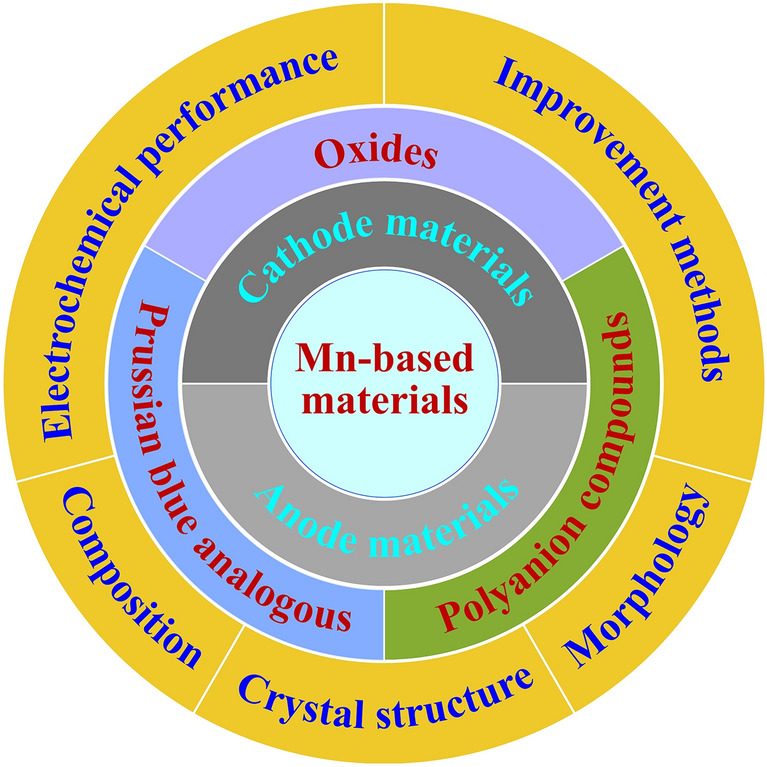

## Introduction

Solar energy, wind energy and other renewable energy are growing quickly and become progressively more important. Due to the intermittent nature of solar energy and wind energy, energy storage systems are needed to store energy, stable the modern grid and supply round-the-clock power [1, 2]. The development of energy storage systems will drive the growth of renewable energy. Among various energy storage systems, battery energy storage systems based on rechargeable batteries (secondary batteries) have attracted great interest because of their high conversion efficiency, flexibility and simple maintenance [3–6]. Lithium-ion batteries (LIBs) have been developed and are now playing more important role in our lives. Large-scale LIBs have attracted enormous attention and been considered one of the most promising energy storage systems [7–9]. However, the limited resources and growing price of lithium resources hinder LIBs applications in large-scale energy storage.

Sodium-ion batteries (SIBs) are strong candidates for large-scale energy storage because of abundant sodium resources and low cost. Up to now, various materials have been investigated for SIBs including cathode, anode and electrolyte materials [10–16]. Nevertheless, safety issue of organic electrolytes is a much notable realistic factor affecting commercialization. Compared with organic electrolytes, aqueous electrolytes are safer and eco-friendly. Furthermore, the ionic conductivity of aqueous electrolyte is larger by almost two orders of magnitude than that of organic electrolytes [8, 17]. Therefore, aqueous SIBs based on aqueous electrolytes have attracted intensive attention for large-scale energy storage, because of their high safety, low cost, convenient manufacture, environment friendliness and easy recycle [8, 18–21]. However, the electrochemical window of aqueous electrolytes is much narrower than that of organic electrolytes. The stable electrochemical window of water is approximately 1.23 V, beyond which water electrolysis will occur with O_2_ or H_2_ gas evolution. Hence, the working potential of electrode materials must be located between H_2_ and O_2_ evolution potentials, which leads to lower energy density of aqueous batteries. To widen the electrochemical window of aqueous electrolytes, many researches have been devoted to optimizing the electrolytes and developing high-voltage electrolytes [22–25]. Hou et al. added surfactant (sodium dodecyl sulfate) to aqueous electrolyte and expanded the electrochemical stability window to about 2.5 V [22]. Tomiyasu et al. reported a saturated sodium perchlorate aqueous solution with a potential window of approximately 3.2 V [23]. In addition, the developed “water-in-salt” electrolytes could expand the electrochemical window to 3 V [24, 25].

The electrochemical performance of aqueous SIBs has also been influenced by electrode materials. The insertion/extraction reactions of Na ions in aqueous electrolytes are more complicated, and thereby affecting the selectivity of electrode materials. The chemical stability of electrode materials is also very important in aqueous electrolyte system. The cycling stability of electrode materials will be affected by side reactions on the electrode surface with H_2_O or residual O_2_ [20]. For exploring suitable electrode materials, the chemical stability, elemental abundance, charge transfer number, redox potentials and electronic conductivity should be considered. Up to now, various electrode materials have been developed for aqueous SIBs, including manganese-based oxides, vanadium-based oxides, Prussian blue analogues, polyanion compounds and organic materials [8, 20, 21, 26–29]. Among them, Mn-based electrode materials have attracted tremendous interests because of abundant reserves of manganese, low cost, low toxicity, rich valence states of manganese and interesting electrochemical performance [27, 29–33]. Mn has multiple oxidation states, such as Mn^2+^, Mn^3+^, Mn^4+^, Mn^6+^ and Mn^7+^, and the redox reactions of Mn^4+^/Mn^3+^ and Mn^4+^/Mn^2+^ can provide high redox potential and one or two-electron transfer, which will lead to high specific capacity as well as high energy density. For MnO_2_ materials, their theoretical capacity is 308 mAh g^− 1^ based on one-electron transfer and 617 mAh g^− 1^ based on two-electron transfer [34, 35]. Tarascon et al. first reported the electrochemical reaction of sodium with *λ*-MnO_2_ in 1 M NaClO_4_ in propylene carbonate [36]. They found that Na reaction with *λ*-MnO_2_ induced an irreversible phase transformation to Na_*x*_MnO_2_, which could reversibly cycle 0.6 Na atom for each Mn atom. In aqueous electrolyte of 1 M Na_2_SO_4_, *λ*-MnO_2_ cathode material showed excellent energy storage functionality with a specific capacity of ~ 80 mAh g^− 1^ [37]. Using 7 M NaOH solution as electrolyte, *γ*-MnO_2_ cathode material displayed a discharge capacity of 225 mAh g^− 1^ [38]. However, the reversible capacity of MnO_2_ cathode material was still lower than its theoretical capacity in aqueous electrolyte. Except for MnO_2_ materials, Na_0.44_MnO_2_, as a promising cathode material, has been widely investigated for aqueous SIBs because of low cost and high theoretical capacity (120 mAh g^−1^) [39]. However, Na_0.44_MnO_2_ electrode usually showed a reversible capacity lower than 50 mAh g^− 1^ in aqueous electrolyte [40, 41]. In addition, Mn-based Prussian blue analogues and polyanion compounds have also been reported. Sun et al. investigated Na_2_MnFe(CN)_6_ electrode in 1 M Na_2_SO_4_ aqueous solution and obtained a reversible capacity of about 85 mAh g^− 1^ [42]. Gao et al. reported Na_3_MnTi(PO_4_)_3_ material as cathode and anode, and a symmetric cell based on 1 M Na_2_SO_4_ electrolyte exhibited a reversible capacity of 57.9 mAh g^− 1^ [43]. Different Mn-based electrode materials presented various electrochemical performance.

**Fig. 1:**
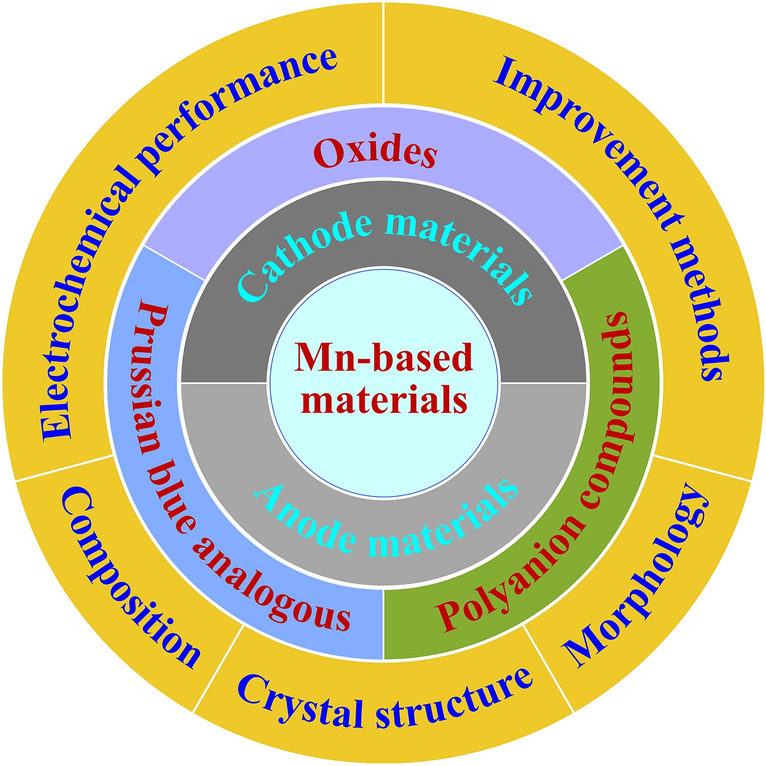
Outline of the Mn-based electrode materials for aqueous SIBs

In order to improve the electrochemical performance of Mn-based electrode materials in aqueous electrolyte, some effective improvement methods, such as electrolyte optimization, element doping, morphology optimization and carbon modification, have been proposed. For example, by adding 2 M MgSO_4_ to Na_2_SO_4_ solution, the reversible capacity of *δ*-MnO_2_ electrode in Na_2_SO_4_ solution increased from 40 to 100 mAh g^− 1^ [44]. With calcium doping, rate capability of calcium-doped Na_0.4_MnO_2_ electrode in 1 M NaClO_4_ solution was enhanced (43% capacity increase at 50C rate) [45]. Using reduced graphene oxide to modify Na_2_MnFe(CN)_6_ material, the discharge capacity of Na_2_MnFe(CN)_6_ electrode increased from 71.0 to 115.4 mAh g^− 1^, and its cycling performance was also improved [46]. To date, different Mn-based electrode materials have been investigated for aqueous SIBs and their electrochemical performance has been improved. In this review, we give an overview about Mn-based electrode materials (both cathodes and anodes) for aqueous SIBs, including oxides, Prussian blue analogues and polyanion compounds. Figure 1 shows the contents. We summarize and discuss the composition, crystal structure, morphology, electrochemical properties and improvement methods. We believe this review is helpful to understand and develop Mn-based electrode materials for aqueous SIBs.

## Mn-Based Cathode Materials

Various Mn-based materials have been reported as cathode materials for aqueous SIBs, including oxides, Prussian blue analogues and polyanion compounds. Different Mn-based cathode materials exhibit diverse electrochemical performance.

### Oxides

Among Mn-based cathode materials for aqueous SIBs, oxides are the most common materials, including MnO_2_, Mn_5_O_8_, Na_*x*_MnO_2_, Na_x_Mn_y_Ti_1-y_O_2_, etc. Different oxides possess various crystal structures and show diverse electrochemical performance. In this section, different Mn-based oxides will be introduced and improvement methods of electrochemical performance are also summarized.

#### ***MnO***_***2***_

MnO_2_ has been widely studied as a cathode material for rechargeable batteries. MnO_2_ possesses several crystallographic structures, including *α*, *β*, γ, *δ* and *λ* crystal structures. The *α*-, *β*- and γ-MnO_2_ show 1D tunnel structure, the *δ*-MnO_2_ exhibits 2D layered structure, and the *λ*-MnO_2_ possesses 3D spinel structure [47, 48]. MnO_6_ octahedra are the basic units for constructing these crystal structures of MnO_2_ via sharing corners and/or edges. These crystal structures possess different gaps of tunnels or interlayers, which affect intercalation/deintercalation of alkali cations in MnO_2_ lattice [48]. The electrochemical performance of MnO_2_ with different crystal structures has also been investigated in aqueous SIBs.

##### Electrochemical Performance

**Fig. 2:**
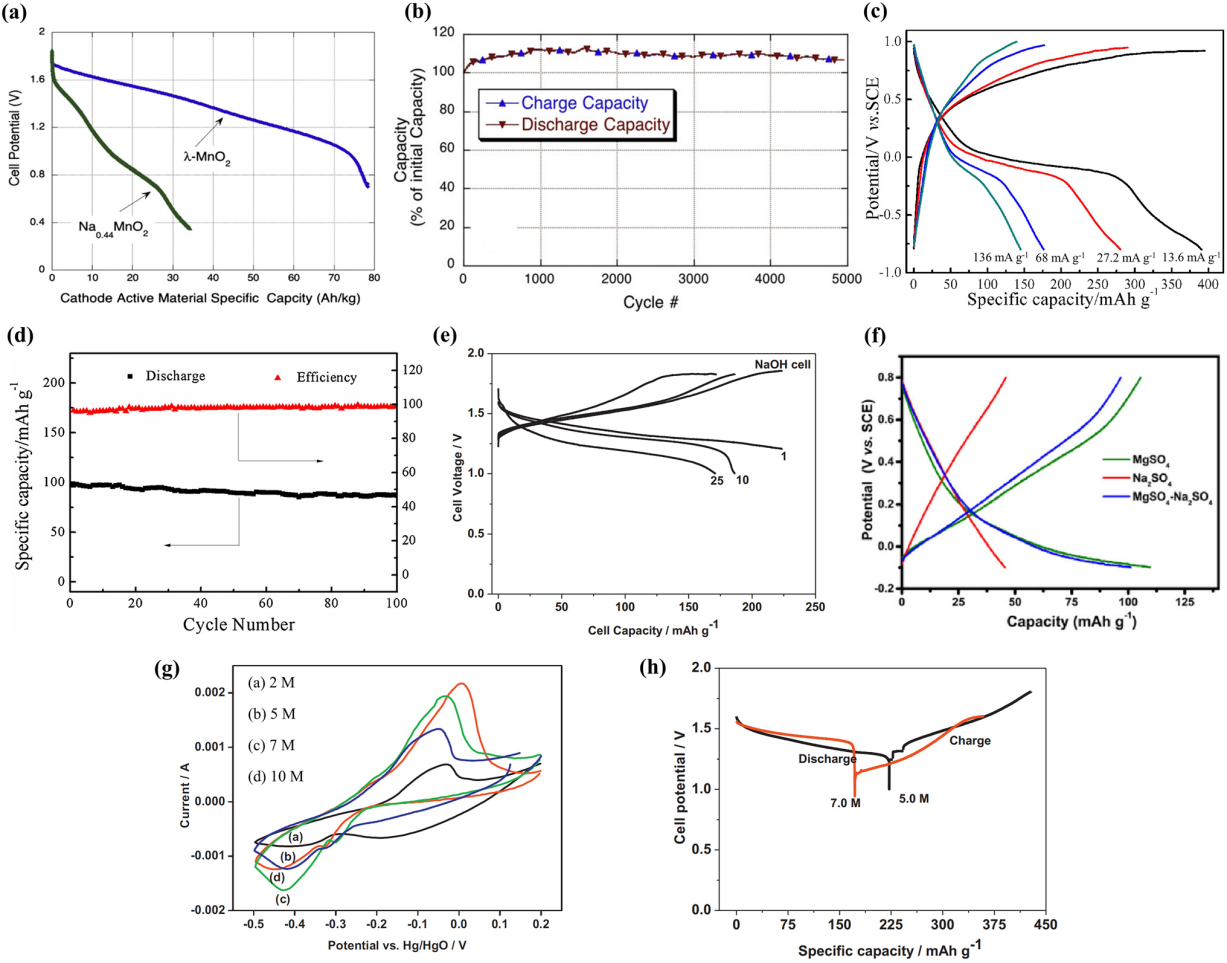
**a** Discharge curves of *λ*-MnO_2_ and Na_4_Mn_9_O_18_ in 1 M Na_2_SO_4_ solution. **b** Charge–discharge cycle testing of a thin *λ*-MnO_2_ electrode cell up to 5000 cycles [37]. Copyright 2012, Elsevier. **c** Charge and discharge curves for *λ*-MnO_2_ electrode in 1 M Na_2_SO_4_ solution at different current densities. **d** The cycling performance of an*λ*-MnO_2_|Na_2_SO_4_|AC capacitor battery at 136 mA g^− 1^ [49]. Copyright 2014, Elsevier. **e** Cyclability of a γ-MnO_2_|NaOH|Zn cell using 7 M NaOH electrolyte [38]. Copyright 2012, Elsevier. **f** Charge and discharge curves of *δ*-MnO_2_ electrode in different electrolytes: 1 M Na_2_SO_4_, 2 M MgSO_4_ and 1 M Na_2_SO_4_-2 M MgSO_4_ solutions [44]. Copyright 2019, Elsevier. **g** CV curves of γ-MnO_2_ electrode in 2, 5, 7 and 10 M NaOH solutions. **h** Discharge–charge profiles of γ-MnO_2_|Zn cells using 5 M and 7 M NaOH solutions [50]. Copyright 2013, Elsevier

MnO_2_ with different crystal structures displays various electrochemical performance. Whitacre et al. investigated the electrochemical performance of *λ*-MnO_2_ material in 1 M Na_2_SO_4_ solution using activated carbon (AC) as anode [37]. Figure 2a shows the discharge curve of *λ*-MnO_2_ electrode, and the discharge capacity is about 80 mAh g^− 1^. The *λ*-MnO_2_ electrode had high specific capacity (twofold increase) and specific energy (threefold increase) compared with the Na_4_Mn_9_O_18_ electrode. A thin *λ*-MnO_2_/AC full cell (electrode thickness of < 100 µm) exhibited outstanding cycling performance, and there was no loss in initial capacity for 5000 cycles, as shown in Fig. 2b. The stability of *λ*-MnO_2_ cathode material might result from the introduction of proton or hydroxide species into the lattice, the stability of H_2_O/MnO_2_ interface, and stable AC anode material. Furthermore, using graphite sheet as counter electrode, a high initial discharge capacity of 390.7 mAh g^− 1^ at 13.6 mA g^− 1^ was obtained for *λ*-MnO_2_ electrode in 1 M Na_2_SO_4_ solution (Fig. 2c) [49]. The discharge capacity was higher than theoretical capacity (308 mAh g^− 1^), which could result from the surface adsorption–desorption of Na ions resulting in slight capacitive behavior during the initial charge–discharge. However, using AC as anode, a *λ*-MnO_2_|Na_2_SO_4_|AC capacitor battery showed only a discharge capacity of 115.3 mAh g^− 1^ at 68 mA g^− 1^, and the capacity retention was more than 90% after 100 cycles (Fig. 2d).

Apart from *λ*-MnO_2_, γ-MnO_2_ cathode material was investigated by Minakshi using Zn as anode and 7 M NaOH solution as electrolyte [38]. A γ-MnO_2_|NaOH|Zn cell displayed a discharge capacity of 225 mAh g^− 1^ at 8 mA g^− 1^, and the discharge capacity decreased to 171 mAh g^− 1^ at 25th cycle (Fig. 2e). There was 24% capacity loss after 25 cycles, and the capacity degradation could be attributed to the anodic dissolution of Zn and the MnO_2_ electrode incorporating some Zn during discharge processes, which could inhibit the sodium intercalation.

In addition, *δ*-MnO_2_ cathode material was studied in three different electrolyte solutions (1 M Na_2_SO_4_, 2 M MgSO_4_ and 1 M Na_2_SO_4_-2 M MgSO_4_) using AC as counter electrode [44]. As shown in Fig. 2f, the reversible capacity of *δ*-MnO_2_ in 1 M Na_2_SO_4_ solution was about 40 mAh g^− 1^ at 200 mA g^− 1^, and the reversible capacity increased to 100 mAh g^− 1^ for 1 M Na_2_SO_4_-2 M MgSO_4_ solution, which suggested that the presence of MgSO_4_ in the electrolyte changed dramatically the electrochemical performance of *δ*-MnO_2_.

From the above discussion, it is clear that the crystal structure of MnO_2_ has a great impact on its reversible capacity and cycling performance. Among various crystal structures, *λ*-MnO_2_ material exhibited better electrochemical performance. In addition, counter electrode and aqueous electrolyte also affected the electrochemical performance of MnO_2_ electrode.

##### Improvement Methods

MnO_2_, especially γ-MnO_2_ and *δ*-MnO_2_, presented low electrochemical performance in aqueous electrolyte, which was needed to be further improved. Electrolyte optimization and element doping have been adopted to improve the electrochemical performance of MnO_2_ electrode materials.

1. **Optimization of Electrolyte**

The electrochemical performance of MnO_2_ could be improved by optimizing aqueous electrolytes. Minakshi and Meyrick investigated the effect of NaOH concentration on the electrochemical performance of γ-MnO_2_ cathode [50]. Cyclic voltammetric (CV) curves of γ-MnO_2_ electrode in NaOH solutions with various concentrations (2, 5, 7 and 10 M) are shown in Fig. 2g, and the best electrochemical performance was obtained in 7 M NaOH solution. The discharge capacities of MnO_2_|Zn cells were 220 and 170 mAh g^− 1^ for 5 and 7 M NaOH solutions (Fig. 2h), respectively. After 40 cycles, the capacity retention for both the MnO_2_|Zn cells was more than 90%. As a result, the electrochemical performance of γ-MnO_2_ cathode could be optimized by increasing NaOH concentration, and the highest reversible capacity was obtained in 5 M NaOH solution electrolyte.

Except for optimizing electrolyte concentration, electrolyte additive was also adopted to improve electrochemical performance. Liu et al. presented the effect of MgSO_4_ addition into Na_2_SO_4_ solution on the electrochemical performance of *δ*-MnO_2_ cathode [44]. With the addition of 2 M MgSO_4_, the specific capacity of *δ*-MnO_2_ cathode increased from 40 to 100 mAh g^− 1^ (Fig. 2f). The improvement could be attributed to the reversible co-intercalation of Na^+^ and Mg^2+^, which is similar to the storage mechanism of Na-Mg hybrid battery. In co-intercalation-type Na-Mg hybrid battery, Na^+^ and Mg^2+^ dual-ion electrolyte was adopted, and Na^+^ and Mg^2+^ ions could be intercalated/deintercalated into cathode [51]. Therefore, optimizing the aqueous electrolyte can enhance effectively the electrochemical performance of MnO_2_ electrodes.

(2) **Element Doping or Incorporating**

**Fig. 3:**
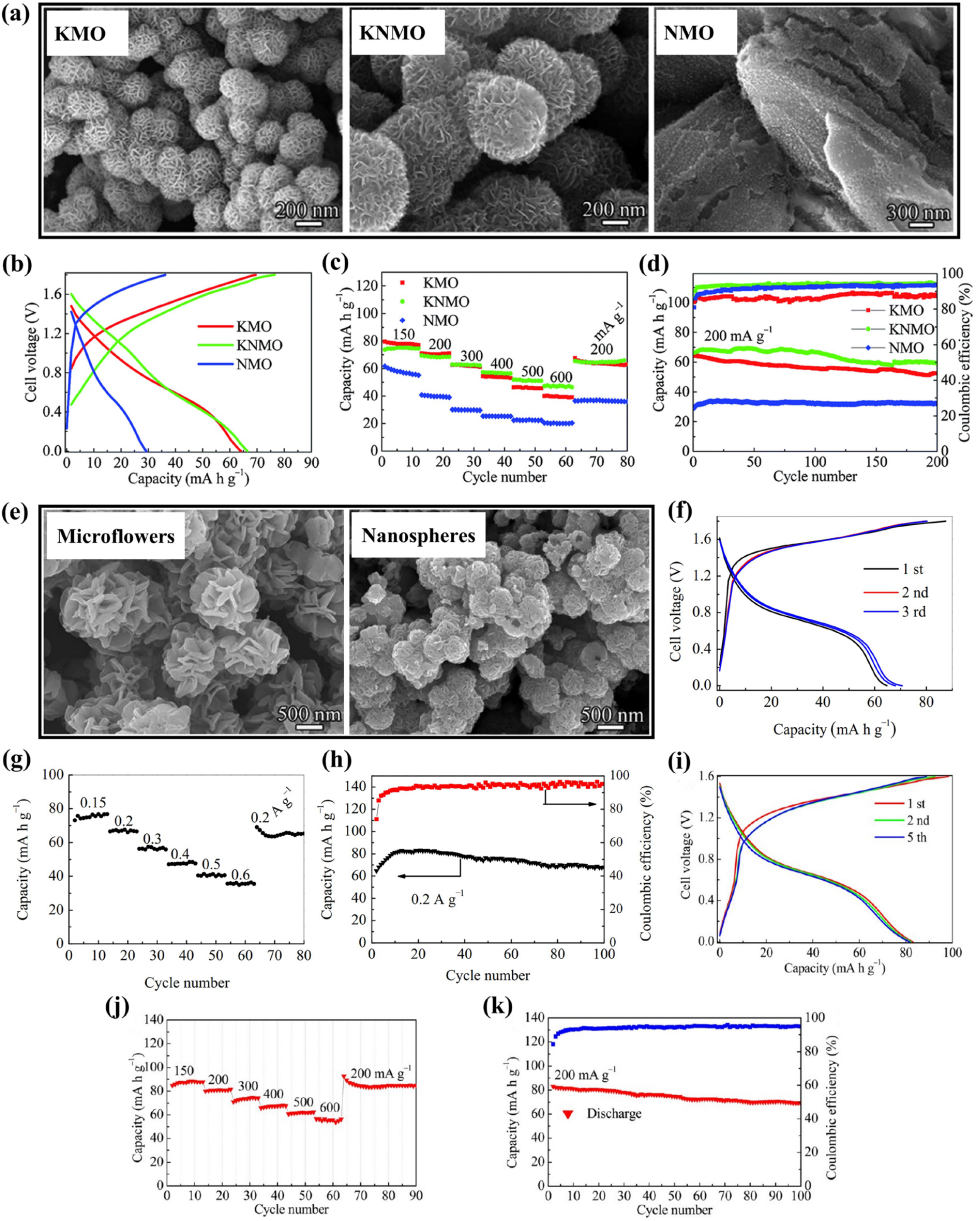
**a** SEM (scanning electron microscope) images of KMO, KNMO and NMO. **b** Charge/discharge profiles (at 200 mA g^− 1^), **c** Capacity retention at different current densities and **d** Cycling performance (at 200 mA g^− 1^) of NaTi_2_(PO_4_)_3_|KMO, NaTi_2_(PO_4_)_3_|KNMO and NaTi_2_(PO_4_)_3_|NMO full cells [52]. Copyright 2015, Royal Society of Chemistry. **e** SEM images of hierarchical layered K_0.27_MnO_2_ microflowers and hollow K_0.27_MnO_2_ nanospheres. **f** Charge/discharge profiles (at 200 mA g^− 1^), **g** Capacity retention at different current densities and **h** Cycling performance (at 200 mA g^− 1^) of NaTi_2_(PO_4_)_3_|K_0.27_MnO_2_ microflowers full cell. [53]. Copyright 2014, Elsevier. **i** Charge/discharge profiles (at 200 mA g^− 1^), **j** Capacity retention at different current densities and **k** Cycling performance (at 200 mA g^− 1^) of NaTi_2_(PO_4_)_3_|K_0.27_MnO_2_ nanospheres full cell [54]. Copyright 2016, American Chemical Society

Element incorporating was adopted to improve the electrochemical performance of MnO_2_. Nanostructured *δ*-MnO_2_ incorporated with K and Na ions was investigated by Liu et al. [52]. The synthesized (K, Na)-incorporated *δ*-MnO_2_ materials were K_0.34_MnO_2_ (KMO), Na_0.56_MnO_2_ (NMO) and K_0.15_Na_0.26_MnO_2_ (KNMO), which had different morphologies, as shown in Fig. 3a. The reversible discharge capacities were 64, 30 and 66.4 mAh g^− 1^ at 200 mA g^− 1^ for KMO, NMO and KNMO electrodes in 1 M Na_2_SO_4_ solution using NaTi_2_(PO_4_)_3_ as anodes (Fig. 3b), respectively. The incorporation of K and Na ions into *δ*-MnO_2_ affected greatly the electrochemical properties of layered *δ*-MnO_2_, and the KNMO and KMO electrodes not only had high capacity but also exhibited superior rate capability (Fig. 3c). The KNMO electrode showed superior reversible capacity and outstanding cycling stability with 90% capacity retention (Fig. 3d). The excellent electrochemical performance of the KNMO and KMO nanospheres could be attributed to adequate crystallinity and hierarchical structure.

In addition, hierarchical layered K_0.27_MnO_2_ microflowers and hollow K_0.27_MnO_2_ nanospheres (Fig. 3e) were also investigated in 1 M Na_2_SO_4_ solution using NaTi_2_(PO_4_)_3_ as anode [53, 54]. A full cell with K_0.27_MnO_2_ microflowers attained an initial discharge capacity of 64.7 mAh g^− 1^ at 200 mA g^− 1^ (Fig. 3f), and exhibited excellent rate capability (Fig. 3g) and long cyclic life without capacity loss after 100 cycles (Fig. 3h). Compared with the full cell with K_0.27_MnO_2_ microflowers, a full cell assembled using hollow K_0.27_MnO_2_ nanospheres demonstrated a high initial discharge capacity of 83 mAh g^− 1^ at 200 mA g^− 1^ (Fig. 3i). The full cell with hollow K_0.27_MnO_2_ nanospheres also displayed excellent rate performance (Fig. 3j) and high cyclic stability up to 100 cycles with 83% capacity retention (Fig. 3k). By comparing Fig. 3b, f, it could be found that when changing K_0.34_MnO_2_ to K_0.27_MnO_2_, voltage plateaus in the charge and discharge curves increased, but the reversible capacities were almost the same. From Fig. 3e to Fig. 3k, it could be concluded that the particle size and morphology of K_0.27_MnO_2_ particles affected the electrochemical performance of K_0.27_MnO_2_ electrode. Thus, the electrochemical performance of (K, Na)-incorporated *δ*-MnO_2_ materials could be significantly enhanced by optimizing incorporation content of K and Na ions and tuning their particle size and morphology.

**Fig. 4:**
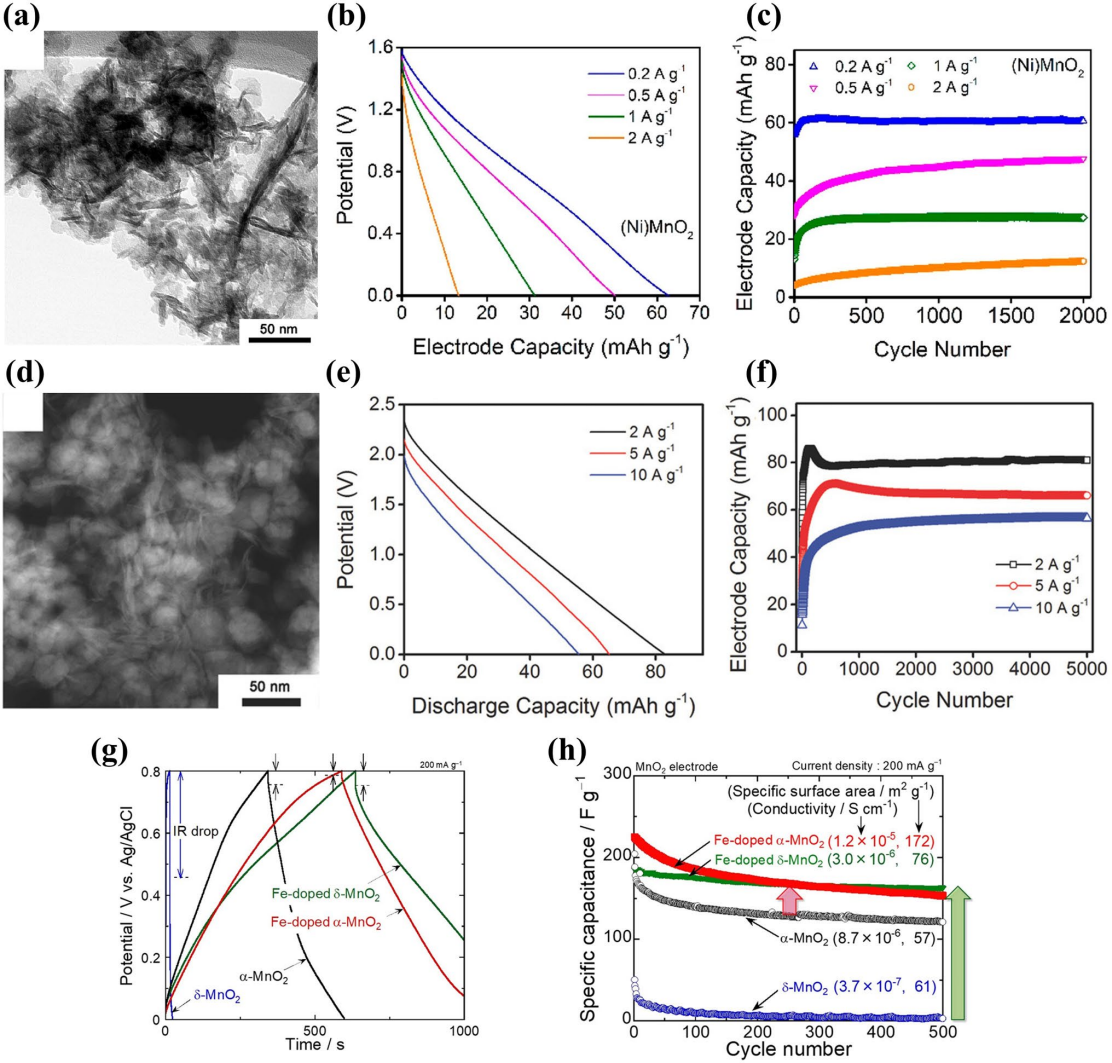
**a** TEM image of (Ni)MnO_2_ nanosheets. **b** Discharge curves of (Ni)MnO_2_ electrode at various current densities. **c** Cycling performance of (Ni)MnO_2_ electrode at various current densities [55]. Copyright 2019, American Chemical Society. **d** TEM image of Co-Mn–O nanomaterials. **e** Discharge curves of biphase Co-Mn–O electrode at various current densities. **f** Cycling performance of biphase Co-Mn–O electrode at various current densities [59]. Copyright 2017, Wiley–VCH. **g** Charge–discharge curves for MnO_2_ and Fe-doped MnO_2_ electrodes in 0.5 M Na_2_SO_4_ solution at the second cycle. **h** Dependence of specific capacitance on cycle number for MnO_2_ and Fe-doped MnO_2_ electrodes [60]. Copyright 2020, American Chemical Society

Apart from element incorporating, doping of Ni, Co and Fe ions into MnO_2_ has also been investigated to enhance its electrochemical performance. Shan et al. reported a framework Ni-doped *δ*-MnO_2_ ((Ni)MnO_2_) material as cathode material [55]. The (Ni)MnO_2_ nanosheets were synthesized by wet chemistry method, and their transmission electron microscopy (TEM) image was shown in Fig. 4a. A symmetric full cell assembled with (Ni)MnO_2_ electrodes and 1 M Na_2_SO_4_ solution as electrolyte delivered a discharge capacity of 63 mAh g^− 1^ at 200 mA g^− 1^, and superior cycle stability without capacity loss over 2000 cycles at 200–2000 mA g^− 1^ (Fig. 4b-c). The storage of Na ions in (Ni)MnO_2_ electrode was a single-phase solid-solution reaction. The pseudocapacitive Na-ion storage, which was promising for high-rate performance [56–58], was enhanced by the framework Ni-doping which formed solid-solution type layered structure with disordered [NiO_6_] octahedra in intralayer framework of ordered [MnO_6_] octahedra. Similar to Ni doping, Co-doped MnO_2_ was also investigated by Shan et al. [59]. However, a framework Co-doped *δ*-MnO_2_ material was unable to be formed by wet chemistry method, and the synthesized material was biphase cobalt-manganese oxide (Co-Mn–O) comprised of (Co_0.83_Mn_0.13_Va_0.04_)_tetra_(Co_0.38_Mn_1.62_)_octa_O_3.72_ (Va: vacancy; tetra: tetrahedral sites; octa: octahedral sites) spinel phase and MnO_2_⋅H_2_O birnessite phase. As shown in Fig. 4d, the synthesized Co-Mn–O nanomaterials had two distinct morphologies: nanoparticle and 2D nanosheet. The biphase Co-Mn–O material displayed also excellent electrochemical performance, and a symmetric full cell based on biphase Co-Mn–O electrodes and 1 M Na_2_SO_4_ electrolyte demonstrated a reversible discharge capacity of 81 mAh g^− 1^ at 2000 mA g^− 1^, high rate performance (57 mAh g^− 1^ at 10,000 mA g^− 1^) (Fig. 4e) and long-term cycling stability (no obvious capacity degradation over 5000 cycles) (Fig. 4f). The improved electrochemical performance of the Co-Mn–O material could be attributed to the synergistic interaction between spinel phase and birnessite phase and the vacancy of the tetrahedral sites of spinel phase.

Furthermore, Usui et al. investigated the effect of Fe doping on the electrochemical performance of MnO_2_ electrodes in aqueous Na_2_SO_4_ solution [60]. Compared with MnO_2_ electrodes, the Fe-doped MnO_2_ electrodes exhibited higher reversible capacity (Fig. 4g). The Fe doping into MnO_2_ reduced the IR drop and enhanced the capacity, and in particular, the IR drop reduction and capacity increase for *δ*-MnO_2_ electrode were significant because of the improvement in the conductivity (from 3.7 × 10^–7^ to 3.0 × 10^–6^ S cm^–1^ with 11 at% Fe doping). Figure 4h gives the cycling performance of *α*-MnO_2_, *δ*-MnO_2_, Fe-doped *α*-MnO_2_ and Fe-doped *δ*-MnO_2_ electrodes. In the initial 100 cycles, the Fe-doped *α*-MnO_2_ electrode had the highest capacitance. The Fe-doped *δ*-MnO_2_ electrode exhibited the best cycling performance with the capacity retention of 87% after 500 cycles. Accordingly, element doping or incorporating is a very effective approach to improve the electrochemical performance of MnO_2_ electrode materials.

In summary, MnO_2_ materials are suitable to be used as cathode materials for aqueous SIBs, and their electrochemical performance can be effectively improved by optimizing electrolyte, element doping or incorporating, and tuning particle size and morphology. The electrochemical properties of MnO_2_ materials mentioned above are summarized in Table 1.

**Table 1:** Electrochemical properties of the Mn-based oxides for aqueous SIBs

Working electrode	Counter electrode	Electrolyte	Voltage range (V)	Capacity (mAh g^− 1^) Rate (mA g^− 1^)	Capacity retention (%) (cycles)	Refs.
*λ*-MnO_2_	AC	1 M Na_2_SO_4_	0.7–1.8	80 (-)	No decay (5000)	[37]
*λ*-MnO_2_	Graphite	1 M Na_2_SO_4_	− 0.8–1.0	390.7 (13.6)	59 (500)	[49]
*λ*-MnO_2_	AC	1 M Na_2_SO_4_	0.1–2.2	115.3 (68)	90 (100)	[49]
γ-MnO_2_	Zn	7 M NaOH	1.0–1.8	225 (8)	76 (25)	[38]
γ-MnO_2_	Zn	5 M NaOH	1.0–1.8	220 (8)	90 (40)	[50]
*δ*-MnO_2_	AC	1 M Na_2_SO_4_	− 0.1–0.8	40 (200)	No decay (100)	[44]
*δ*-MnO_2_	AC	1 M Na_2_SO_4_ − 2 M MgSO_4_	− 0.1–0.8	100 (200)	86 (100)	[44]
K_0.15_Na_0.26_MnO_2_	NaTi_2_(PO_4_)_3_	1 M Na_2_SO_4_	0–1.8	66.4 (200)	90 (200)	[52]
K_0.34_MnO_2_	NaTi_2_(PO_4_)_3_	1 M Na_2_SO_4_	0–1.8	64 (200)	81 (200)	[52]
K_0.27_MnO_2_	NaTi_2_(PO_4_)_3_	1 M Na_2_SO_4_	0–1.8	64.7 (200)	No decay (100)	[53]
K_0.27_MnO_2_	NaTi_2_(PO_4_)_3_	1 M Na_2_SO_4_	0–1.6	83 (200)	83 (100)	[54]
Ni-doped *δ*-MnO_2_	Ni-doped *δ*-MnO_2_	1 M Na_2_SO_4_	0–1.6	63 (200)	No decay (2000)	[55]
Co_*x*_Mn_3-*x*_O_4_-*δ*-MnO2	Co_*x*_Mn_3-*x*_O_4_-*δ*-MnO_2_	1 M Na_2_SO_4_	0–2.5	81 (2000)	No decay (5000)	[59]
Na_0.27_MnO_2_	Pt	0.1 M Na_2_SO_4_	− 0.75–1.25	138 (600)	–	[64]
Na_0.27_MnO_2_	Na_0.27_MnO_2_	0.5 M Na_2_SO_4_	0–2.5	83 (1000)	No decay (5000)	[64]
Na_0.35_MnO_2_	Ni	0.5 M Na_2_SO_4_	0–1.0	43.6 (200)	No decay (5000)	[65]
Na_0.39_MnO_2_	AC	1 M NaClO_4_-0.1 M LiNO_3_	0–0.8	45.1 (60)	90.3 (1000)	[94]
Na_0.44_MnO_2_	Na_0.44_MnO_2_-coated stainless steel	0.5 M Na_2_SO_4_	0.25–0.9	40 (12.1)	–	[40]
Na_0.44_MnO_2_	Graphite	1.5 M NaNO_3_	− 0.1–0.95	48 (60)	94 (90)	[69]
Na_0.44_MnO_2_	Pt	1 M Na_2_SO_4_	0–0.6	43.7 (122.5)	86.2 (100)	[41]
Na_0.44_MnO_2_	NaTi_2_(PO_4_)_3_/C	1 M Na_2_SO_4_	0–1.6	43 (100)	60 (1000)	[72]
Na_0.44_MnO_2_	Zn	1 M Na_2_SO_4_	1.0–1.9	45 (100)	-	[93]
Na_0.44_MnO_2_	Zn	1 M Na_2_SO_4_-0.5 M ZnSO_4_-0.05 M MnSO_4_	1.0–1.9	63 (100)	No decay (170)	[93]
Na_0.44_MnO_2_	Pt	1 M Na_2_SO_4_	-0.3–1.0	77.2 (100)	80 (1000)	[98]
Na_0.44_MnO_2_-CNT	Pt	1 M Na_2_SO_4_	0–0.8	65 (50)	63.4 (300)	[104]
Na_0.58_MnO_2_·0.48H_2_O	Ti	1 M Na_2_SO_4_	-0.1–0.8	80 (80)	No decay (1000)	[83]
Na_0.7_MnO_2.05_	Ti	1 M Na_2_SO_4_	0–0.8	52 (50)	90.1 (600)	[85]
Na_0.95_MnO_2_	Ni	0.5 M Na_2_SO_4_	0–1.0	25.6 (200)	No decay (5000)	[65]
Na_0.95_MnO_2_	Zn	0.5 M CH_3_COONa- 0.5 M Zn(CH_3_COO)_2_	1–2	60	92 (1000)	[86]
NaMnO_2_	Ti	2 M CH_3_COONa	0–1.0	55 (60)	No decay (500)	[88]
Ca_0.07_Na_0.26_MnO_2_	AC	1 M NaClO_4_	-0.1–1.6	40 (-)	98.8 (1000)	[45]
Na_0.5_Mn_0.5_Ti_0.5_O_2_	Desodiated NiHCF	6 M NaClO_4_	0–1.0	46 (30)	95 (100)	[100]
Na_0.66_Mn_0.66_Ti_0.34_O_2_	NaTi_2_(PO_4_)_3_/C	1 M Na_2_SO_4_	0.3–1.7	76 (236)	89 (300)	[99]
Mn_5_O_8_	Mn_5_O_8_	1 M Na_2_SO_4_	0–3.0	116 (5000)	No decay (2000)	[112]
Mn_5_O_8_	Mn_5_O_8_	1 M Na_2_SO_4_	0–3.0	103 (5000)	No decay (5000)	[113]

#### Na_x_MnO_2_

Various Na_*x*_MnO_2_ materials have been studied as cathode materials for aqueous SIBs, including Na_0.27_MnO_2_, Na_0.35_MnO_2_, Na_0.4_MnO_2_, Na_0.44_MnO_2_, Na_0.58_MnO_2_, Na_0.7_MnO_2_, Na_0.95_MnO_2_, NaMnO_2_ and doped/substituted Na_*x*_MnO_2_. These Na_*x*_MnO_2_ cathode materials have different composition, crystal structures and morphologies, which affect their electrochemical performance.

##### Electrochemical Performance

Na_*x*_MnO_2_ can be classified into tunnel-type oxides and layered oxides. With Na content *x* ≥ 0.5, Na_*x*_MnO_2_ presents a two-dimensional layered structure [30, 61, 62]. When *x* ≤ 0.44, Na_*x*_MnO_2_ exhibits a three-dimensional tunnel structure [61, 63].

1. **Na**_***x***_**MnO**_**2**_ ***(x < 0.44)***

**Fig. 5:**
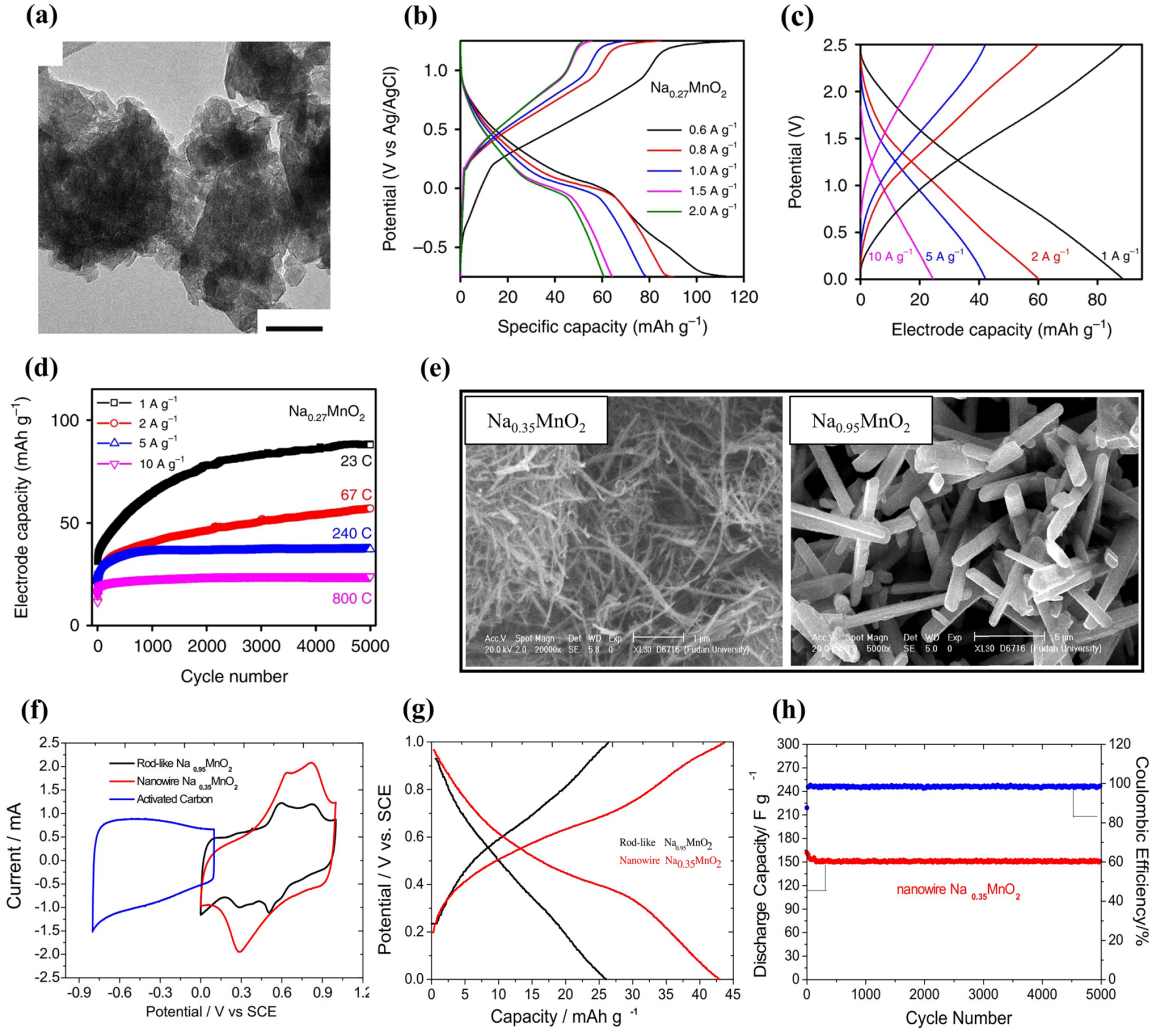
**a** TEM image of Na_0.27_MnO_2_ materials (scale bar, 50 nm). **b** Charge–discharge profiles of Na_0.27_MnO_2_ electrode at 600–2000 mA g^− 1^ (2^nd^ cycle data). **c** Charge–discharge profiles of a symmetric full cell with Na_0.27_MnO_2_ electrodes (after 5000 charge–discharge process). **d** Cycling performance of a symmetric full cell with Na_0.27_MnO_2_ electrodes at various current densities [64]. Copyright 2019, The Authors(s).** e** SEM images of nanowire Na_0.35_MnO_2_ and rod-like Na_0.95_MnO_2_. **f** CV curves of Na_0.35_MnO_2_ and Na_0.95_MnO_2_ electrodes in 0.5 M Na_2_SO_4_ solution at 5 mV s^−1^. **g** Change-discharge curves and** h** Cycling performance of Na_0.35_MnO_2_ and Na_0.95_MnO_2_ electrodes at 200 mA g^−1^ [65]. Copyright 2013, Elsevier

Only a few Na_*x*_MnO_2_ (*x* < 0.44) materials have been reported for aqueous SIBs. A sodium-rich disordered birnessite, Na_0.27_MnO_2_, was reported by Shan et al. [64]. The Na_0.27_MnO_2_ materials synthesized by solid-state method had planar structure (Fig. 5a). Upon water intercalation, Na_0.27_MnO_2_ became Na_0.27_MnO_2_·0.63H_2_O. The Na_0.27_MnO_2_ electrode exhibited an initial discharge capacity of 138 at 600 mA g^− 1^ in 0.1 M Na_2_SO_4_ solution using Pt as counter electrode, and the discharge capacity decreased from 115 to 61 mAh g^− 1^ at 600–2000 mA g^− 1^ with increasing the current density (Fig. 5b). However, a symmetric full cell with Na_0.27_MnO_2_ electrodes and 1 M Na_2_SO_4_ electrolyte showed nearly linear charge–discharge profiles and a discharge capacity of 83 mAh g^− 1^ at 1000 mA g^− 1^ (Fig. 5c). The full cell exhibited excellent cycle stability without obvious capacity loss up to 5000 cycles at various current densities (Fig. 5d). The improved electrochemical performance could be ascribed to Na-rich disordered structure and structural water, as well as co-deintercalation of sodium-ion and hydrated water at high potential charge.

With increasing Na content, Na_0.35_MnO_2_ material was reported. Zhang et al. synthesized nanowire Na_0.35_MnO_2_ by hydrothermal method and investigated its electrochemical performance in 0.5 M Na_2_SO_4_ solution with Ni counter electrode [65]. For comparison, rod-like Na_0.95_MnO_2_ particles were prepared by solid-state reaction, and their SEM images were shown in Fig. 5e. Two separated sharp redox peaks could be observed in CV curves for Na_0.35_MnO_2_ electrode (Fig. 5f), which corresponded to intercalation/deintercalation of sodium ions. In contrast, Na_0.95_MnO_2_ electrode showed two small redox couples, indicating capacitive and pseudocapacitive property. The discharge capacity (43.6 mAh g^− 1^) of Na_0.35_MnO_2_ electrode was higher than that of Na_0.95_MnO_2_ electrode (25.6 mAh g^− 1^) (Fig. 5g), which could be caused by smaller particle size and larger surface area of Na_0.35_MnO_2_ nanowires. The Na_0.35_MnO_2_ electrode also presented excellent cycling performance without capacity degradation after 5000 cycles (Fig. 5h). In addition, a full cell assembled using Na_0.35_MnO_2_ nanowires, polypyrrole (PPy)-coated MoO_3_ (PPy@MoO_3_) nanobelts and 0.5 M Na_2_SO_4_ solution delivered an energy density of 20 Wh kg^− 1^ at 80 W kg^− 1^ and better cycling behavior with only 21% capacity loss after 1000 cycles [66]. Although Na_0.35_MnO_2_ electrode exhibited excellent cycle stability without capacity degradation up to 5000 cycles, its reversible capacity was very much low compared to Na_0.27_MnO_2_ electrode, which could be related to different structure (disordered structure for Na_0.27_MnO_2_) and structural water.

(2) **Na**_***x***_**MnO**_**2**_ ***(x  = 0.44)***

**Fig. 6:**
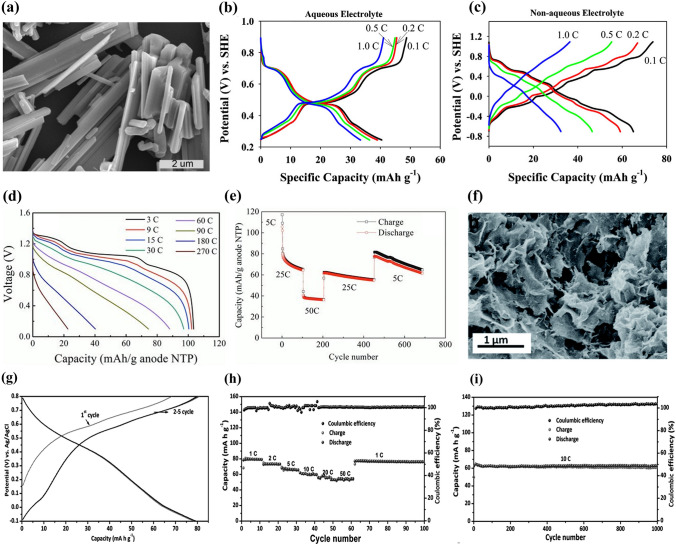
**a** SEM image of the synthesized Na_0.44_MnO_2_ particles. Charge–discharge curves of Na_0.44_MnO_2_ electrode in **b** aqueous electrolyte and **c** non-aqueous electrolyte at different current densities [40]. Copyright 2013, Elsevier. **d** Discharge curves and **e** cycling performance of Na_0.44_MnO_2_|NaTi_2_(PO_4_)_3_ full cell at different rates [73]. Copyright 2013, Wiley‐VCH. **f** SEM image of the synthesized Na_0.58_MnO_2_·0.48H_2_O. **g** Charge–discharge profiles of Na_0.58_MnO_2_·0.48H_2_O electrode at 1C. **h** Rate capability of Na_0.58_MnO_2_·0.48H_2_O electrode. **i** Cycling performance of Na_0.58_MnO_2_·0.48H_2_O electrode at 10C [83]. Copyright 2016, Royal Society of Chemistry

Tunnel-type Na_0.44_MnO_2_ materials have been widely studied as cathode materials for SIBs [67, 68]. Many Na_0.44_MnO_2_ cathode materials have also been reported for aqueous SIBs. Kim et al. investigated the intercalation/deintercalation behavior of sodium ions in Na_0.44_MnO_2_ in both aqueous (0.5 M Na_2_SO_4_) and non-aqueous (1 M NaClO_4_) electrolytes [40]. Rod-shaped Na_0.44_MnO_2_ particles were synthesized by modified Pechini method, and their morphology was shown in Fig. 6a. Only three plateaus in the discharge curves for Na_0.44_MnO_2_ electrode in aqueous electrolyte were observed (Fig. 6b) in comparison with six plateaus for Na_0.44_MnO_2_ electrode in non-aqueous electrolyte (Fig. 6c). This indicated that only part of Na ions could be extracted from Na_0.44_MnO_2_ electrode in aqueous electrolyte. As a result, the discharge capacity of Na_0.44_MnO_2_ electrode in aqueous electrolyte was 40 mAh g^− 1^ at 12.1 mA g^− 1^, which was lower than that in non-aqueous electrolyte (65 mAh g^− 1^). However, the Na_0.44_MnO_2_ electrode displayed enhanced rate capability in aqueous electrolyte with capacity retention of 82% (from 0.1C to 1C), which was higher than that in non-aqueous electrolyte (49%). The differences could be attributed to different apparent diffusion coefficient of Na ions as well as charge transfer resistance and additional resistance from SEI (solid electrolyte interphase) layer.

Besides, in different aqueous electrolytes, the Na_0.44_MnO_2_ electrodes exhibited various electrochemical performance. In 1 M Na_2_SO_4_ solution, Na_0.44_MnO_2_ nanorods demonstrated a reversible discharge capacity of 43.7 mAh g^− 1^ with platinum wire as counter electrode [41]. A reversible discharge capacity of about 50 mAh g^− 1^ at 60 mA g^− 1^ was obtained for Na_0.44_MnO_2_ (rod-like morphology) electrode in 1.5 M NaNO_3_ solution using graphite plate as counter electrode [69]. In 6 M NaOH solution, Na_0.44_MnO_2_ electrode displayed a high reversible capacity of 80.2 mAh g^− 1^ at 0.5C with Zn as counter electrode [70]. This indicated that aqueous electrolytes had a greatly impact on the electrochemical performance of Na_0.44_MnO_2_ electrodes.

Furthermore, some full cells using Na_0.44_MnO_2_ cathodes were also investigated. A novel Na_0.44_MnO_2_|phenazine full cell based on 10 M NaOH solution showed excellent rate capacity and ultralong cycling life with 80% capacity retention after 13,000 cycles [71]. Based on 1 M Na_2_SO_4_ solution, Na_0.44_MnO_2_|NaTi_2_(PO_4_)_3_ full cells also delivered better electrochemical performance [72, 73]. At current rate of 90C, the capacity retention was more than 70% (compared to the capacity at 3C) (Fig. 6d). After 700 cycles at different rates (from 5 to 50C to 5C), the full cell still held about 60% capacity at 5C (compared to the first cycle) (Fig. 6e). The better electrochemical performance could result from good structural stability of anode and cathode in water during high rate electrochemical reaction. Apart from these full cells, similar full cells based on Na_0.44_MnO_2_ cathodes with various anodes, including NaTi_2_(PO_4_)_3_/C [74], wafer-like NaTi_2_(PO_4_)_3_/C [75], frogspawn-like NaTi_2_(PO_4_)_3_/C [76], NaTi_2_(PO_4_)_3_/MWNTs (multiwalled carbon nanotube) [77], NaV_3_(PO_4_)_3_@C nanofiber [78], Na_2_V_6_O_16_·*n*H_2_O [79], PNP@CNT (polyimide-MWCNT composite) [80], amorphous FePO_4_·2H_2_O [81], TiP_2_O_7_ [82], have also been reported, and much improved electrochemical performance has been obtained.

In general, among all the Na_*x*_MnO_2_ materials, tunnel-type Na_0.44_MnO_2_ shows high atmospheric and electrochemical stability [63], leading to high rate capability and better cycling performance, but it usually presents a low specific capacity of less than 80 mAh g^− 1^. Tunnel-type Na_0.44_MnO_2_ is considered as a promising cathode material for aqueous SIBs due to its unique crystal structure and stability.

(3) **Na**_***x***_**MnO**_**2**_ **(*x*** **> 0.5**)

Compared with tunnel-type oxides, layered oxides usually exhibited high specific capacity. Zhang et al. synthesized layered structure Na_0.58_MnO_2_·0.48H_2_O by precipitation method [83]. The Na_0.58_MnO_2_·0.48H_2_O consisted of wrinkled thin sheets (Fig. 6f). A reversible capacity of 80 mAh g^− 1^ was obtained at 1C (80 mA g^− 1^) for Na_0.58_MnO_2_·0.48H_2_O electrode in 1 M Na_2_SO_4_ solution using Ti counter electrode (Fig. 6g). The Na_0.58_MnO_2_·0.48H_2_O electrode exhibited also high rate capability (Fig. 6h) and excellent cycling performance (Fig. 6i). The reversible discharge capacities were 67, 57 and 54 mAh g^− 1^ at 5C, 20C and 50C, respectively, and there was no capacity loss after 1000 cycles. The superior electrochemical performance of Na_0.58_MnO_2_·0.48H_2_O electrode could be attributed to the superior Na-ion storage properties of Na_0.58_MnO_2_·0.48H_2_O and the crystal water in Na_0.58_MnO_2_·0.48H_2_O which could decrease charge transfer resistance and improve the conductance of Na ions.

**Fig. 7:**
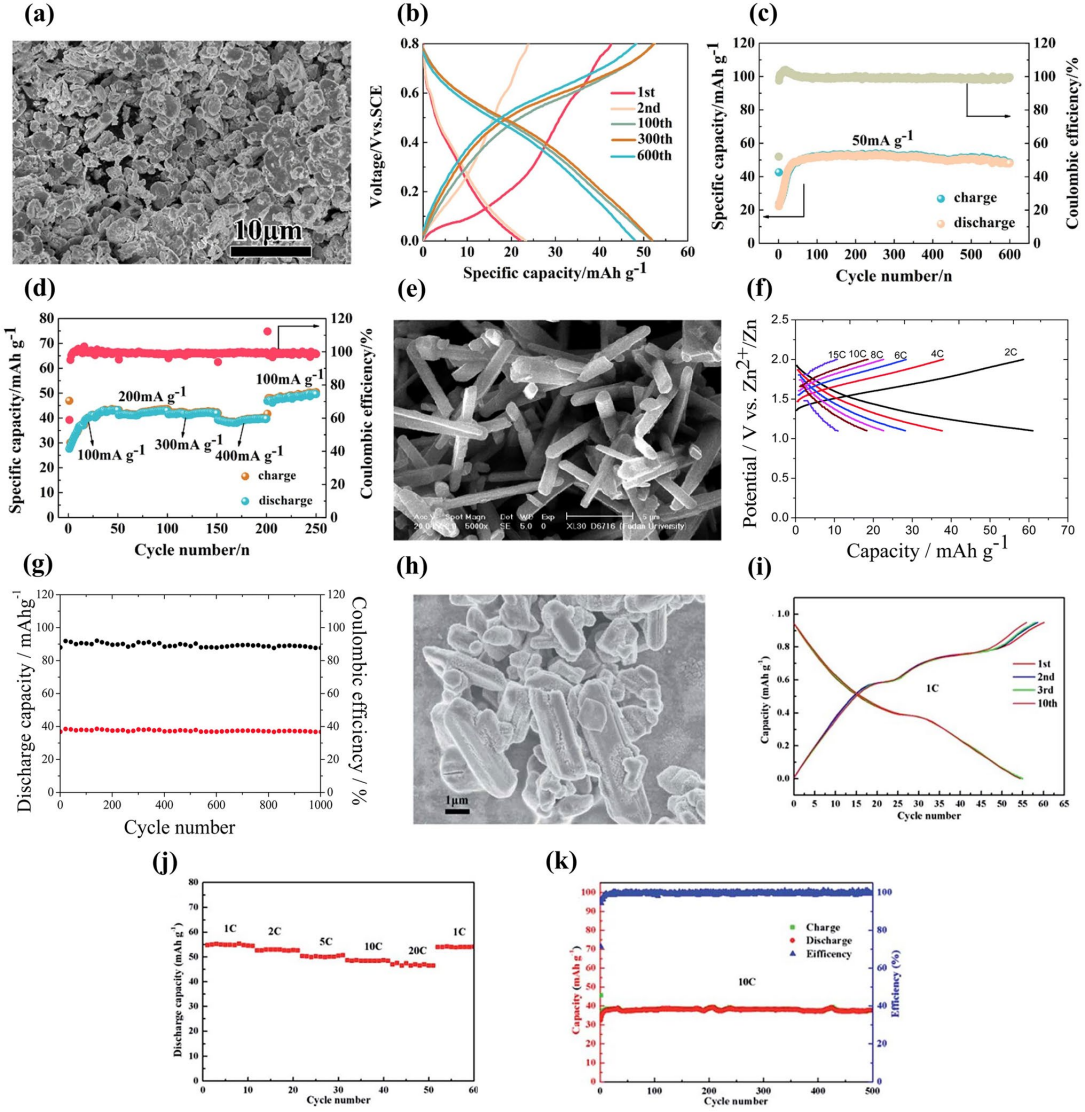
**a** SEM image of the prepared Na_0.7_MnO_2.05_ powders. **b** Charge/discharge profiles at 50 mA g^− 1^, **c** Cycling performance at 50 mA g^− 1^ and **d** Rate performance of the Na_0.7_MnO_2.05_ electrodes in 1 M Na_2_SO_4_ with Ti counter electrode [85]. Copyright 2020, Springer. **e** SEM image of the prepared Na_0.95_MnO_2_ particles. **f** Charge–discharge curves of the rod-like Na_0.95_MnO_2_ electrode at different rates with Zn counter electrode. **g** Cycling performance of a Na_0.95_MnO_2_|Zn full cell at 4C [86]. Copyright 2014, Royal Society of Chemistry. **h** SEM image of the NaMnO_2_. **i** Discharge/charge curves at 1C,** j** Rate capability and **k** Cycling performance at 10C of the NaMnO_2_ electrode [88]. Copyright 2015, Royal Society of Chemistry

Furthermore, Na_0.7_MnO_2_ cathode material was also reported. Rakocevic et al. synthesized 3D tunnel structured Na_0.4_MnO_2_ nanorods (800 °C), hexagonal-layered *α*-Na_0.7_MnO_2.05_ nanoplates (850 °C), and 3D tunnel structured Na_0.44_MnO_2_ powders with rod-like morphology (900 °C) by glycine nitrate method, and investigated their electrochemical behavior in aqueous NaNO_3_ solution using platinum foil as counter electrode [84]. The Na_0.7_MnO_2.05_ electrode showed the highest initial discharge capacity, and the initial discharge capacities were 50, 75 and 46 mAh g^− 1^ for Na_0.4_MnO_2_, Na_0.7_MnO_2.05_ and Na_0.44_MnO_2_, respectively. However, micron-sized Na_0.7_MnO_2.05_ (about 2 µm, as shown in Fig. 7a) prepared by a sol–gel method delivered a low discharge capacity of 22.1 mAh g^− 1^ at 50 mA g^− 1^ in 1 M Na_2_SO_4_ solution (Fig. 7b) [85]. The discharge capacity increased with increasing cycle number and a discharge capacity of 52 mAh g^− 1^ was obtained at the 100th cycle, which could be attributed to the battery activation. After 600 cycles, the Na_0.7_MnO_2.05_ electrode remained 48 mAh g^− 1^ capacity with capacity retention of 90.1% (capacity of the 100th cycle) (Fig. 7c). The Na_0.7_MnO_2.05_ electrode also exhibited good rate performance (Fig. 7d), and the reversible capacities were 42.9, 41.0 and 38.0 mAh g^− 1^ at 200, 300 and 400 mA g^− 1^, respectively. However, the electrochemical performance of Na_0.7_MnO_2.05_ electrode is poor compared to Na_0.58_MnO_2_·0.48H_2_O electrode.

In addition, Na_*x*_MnO_2_ materials with higher sodium content were also investigated for aqueous SIBs. Zhang et al. synthesized rod-like Na_0.95_MnO_2_ particles (Fig. 7e) by solid-state reaction [86]. A full cell Na_0.95_MnO_2_|Zn with 0.5 M CH_3_COONa-0.5 M Zn(CH_3_COO)_2_ electrolyte exhibited a reversible discharge capacity of 60 mAh g^− 1^ at 2C and good cycling performance over 1000 cycles at 4C with only 8% capacity loss, as shown in Fig. 7f-g. The full cell was also regarded as Na-Zn hybrid battery, which used two-component electrolyte (Na^+^ and Zn^2+^ coexisting) [87]. Compared to single ion batteries, the Na-Zn hybrid battery could exhibit high electrochemical performance due to different ion intercalation mechanisms in different electrolytes. Hou et al. reported that NaMnO_2_ cathode material (about 1–2 µm, as shown in Fig. 7h) displayed a discharge capacity of 55 mAh g^− 1^ at 1C (60 mA g^− 1^) in 2 M CH_3_COONa solution using Ti counter electrode (Fig. 7i) [88]. The NaMnO_2_ electrode also showed high rate performance with discharge capacity of 50 mAh g^− 1^ at 10C (Fig. 7j), and good cycling performance without obvious capacity loss after 500 cycles (Fig. 7k). A NaMnO_2_|NaTi_2_(PO_4_)_3_/C full cell gave an energy density of 30 Wh kg^− 1^ at 50 W kg^− 1^ and showed 75% capacity retention after 500 cycles at 5C.

In brief, the Na_*x*_MnO_2_ materials with different sodium content, crystal structure and morphology exhibit various electrochemical performance. Tunnel-type oxides show better cycling performance, and layered oxides display relatively high specific capacity. Among the Na_*x*_MnO_2_ materials, Na_0.44_MnO_2_ materials have been widely investigated and showed better electrochemical performance.

##### Improvement Methods

For practical application, the electrochemical performance of Na_*x*_MnO_2_ materials, especially rate capability and cycling performance, should be further enhanced. Some methods have been adopted to improve the electrochemical performance of Na_*x*_MnO_2_ cathode materials, including electrolyte optimization, morphology optimization, element doping or substitution, and carbon modification.

1. ** Optimization of Electrolyte**

**Fig. 8:**
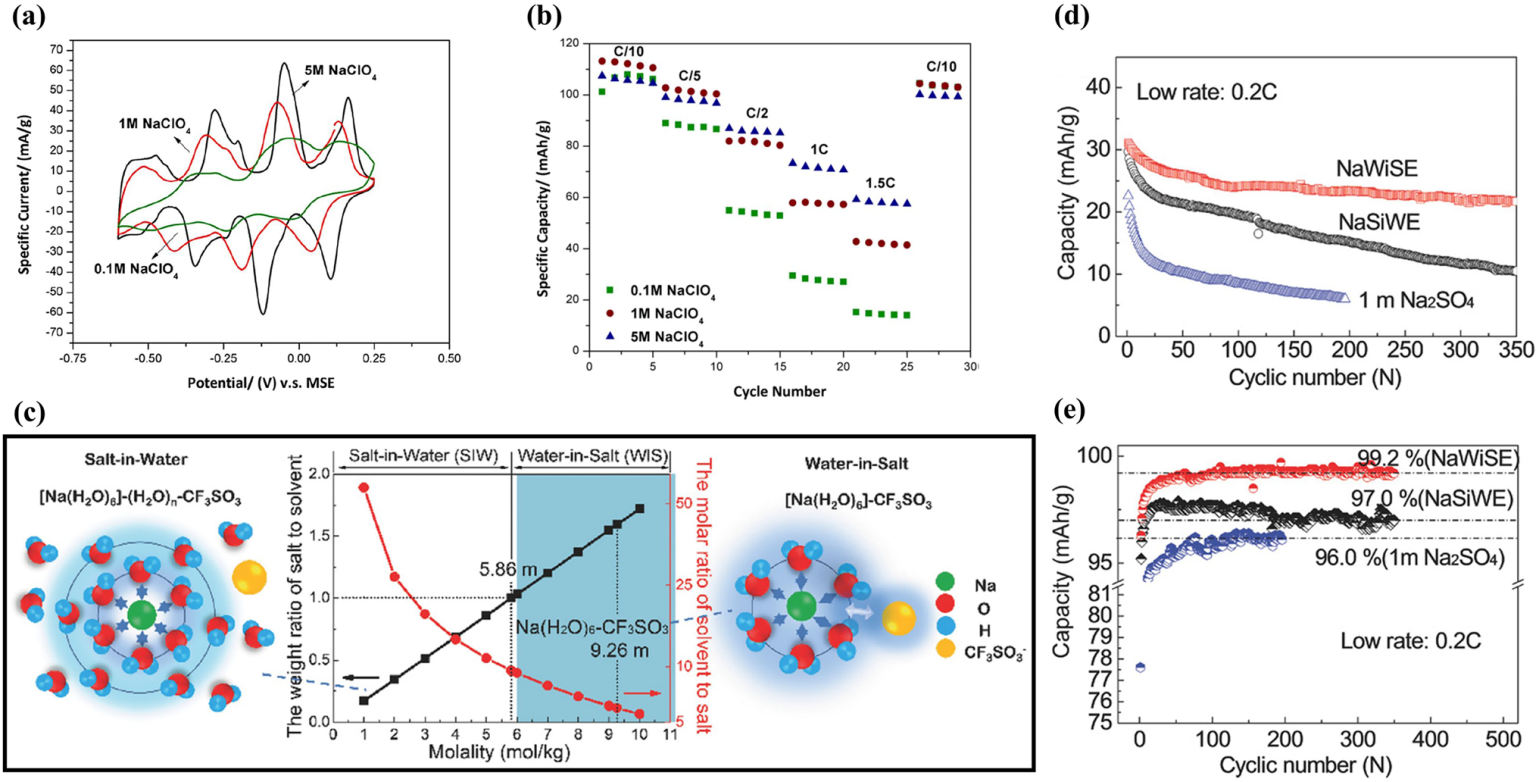
**a** CV curves of Na_0.44_MnO_2_ electrode in 0.1–5 M NaClO_4_ solution at scan rate of 0.1 mV s^− 1^. **b** Rate performance of Na_0.44_MnO_2_|NaTi_2_(PO_4_)_3_ full cell (Na_0.44_MnO_2_ electrode: 100 mg cm^−2^ with thickness of 950 μm; carbon-coated NaTi_2_(PO_4_)_3_ electrode: 113 mg cm^−2^ with thickness of 530 μm) with different NaClO_4_ concentrations: 0.1 M, 1 M and 5 M [89]. Copyright 2015, The Author(s). **c** The molar and weight salt/solvent ratios in NaCF_3_SO_3_-H_2_O binary system. **d** Cycle life and **e** Coulombic efficiency of Na_0.66_Mn_0.66_Ti_0.34_O_2_|NaTi_2_(PO_4_)_3_ full cells in 1 M Na_2_SO_4_, 2 M NaCF_3_SO_3_ (NaSiWE) and 9.26 M NaCF_3_SO_3_ (NaWiSE) electrolytes at 0.2C [92]. Copyright 2017, Wiley‐VCH

First, electrolyte salt concentration was optimized to improve the electrochemical performance of Na_*x*_MnO_2_ cathode. Wu et al. examined the effect of NaClO_4_ concentration on electrochemical performance of a Na_0.44_MnO_2_|NaTi_2_(PO_4_)_3_ full cell [89]. As shown in Fig. 8a, the redox peaks were sharper and closer at higher concentration, which was consistent with ionic conductivity increase of NaClO_4_ electrolyte (the highest ionic conductivity at 5 M). With increasing concentration, the equilibrium potentials shifted to more positive values. The NaClO_4_ concentration affected strongly the discharge capacity, in particularly, at higher rate (Fig. 8b), and the capacity retention at 1.5C was 13.3%, 37.8% and 54.8% (capacity at 0.1C) for 0.1, 1 and 5 M NaClO_4_, respectively. Similarly, high discharge capacity and good capacity retention were obtained for Na_0.44_MnO_2_ cathode in saturated NaClO_4_ solution (not 1 M and 8 M NaClO_4_) because of low Mn dissolution in high concentration electrolyte [90]. High concentration electrolyte improving the electrochemical performance of Na_0.44_MnO_2_ was proved in NaOH aqueous electrolyte [91]. A Na_0.44_MnO_2_|Zn dual-ion battery showed the best rate performance in 6–8 M NaOH solution. When the NaOH concentration exceeded 8 M, the rate performance became poor. The capacity retention after 500 cycles was 27.4%, 33.2%, 54.2%, 64.3% and 65.8% in 1, 3, 6, 8 and 10 M NaOH, respectively. Better cycling stability was obtained in higher NaOH concentration, which could be caused by the reduction of water redox activity and side reactions in high concentration. In addition, Na_0.66_Mn_0.66_Ti_0.34_O_2_ electrode also presented better electrochemical performance in “water-in-salt” electrolyte compared to in “salt-in-water” electrolyte [92]. As shown in Fig. 8c, for Na^+^ in dilute aqueous solutions with salt concentration below 5 M (salt-in-water), its solvation sheath was composed of at least two layers. However, when salt concentration was above 9 M (water-in-salt), the resultant solution could be visualized as a liquefied salt, which could lead to some new properties including transport and interphasial chemistry. As a result, among the full cells Na_0.66_Mn_0.66_Ti_0.34_O_2_|NaTi_2_(PO_4_)_3_ with 1 M Na_2_SO_4_, 2 M NaCF_3_SO_3_ (NaSiWE) and 9.26 M NaCF_3_SO_3_ (NaWiSE) electrolytes, the full cell with NaWiSE displayed the highest capacity, the best cycling stability and the highest coulombic efficiency (Fig. 8d–e). The superior electrochemical performance could be attributed to the formation of Na^+^-conducting SEI, which suppressed the water decomposition. Therefore, optimizing the electrolyte salt concentration can effectively improve the electrochemical performance.

**Fig. 9:**
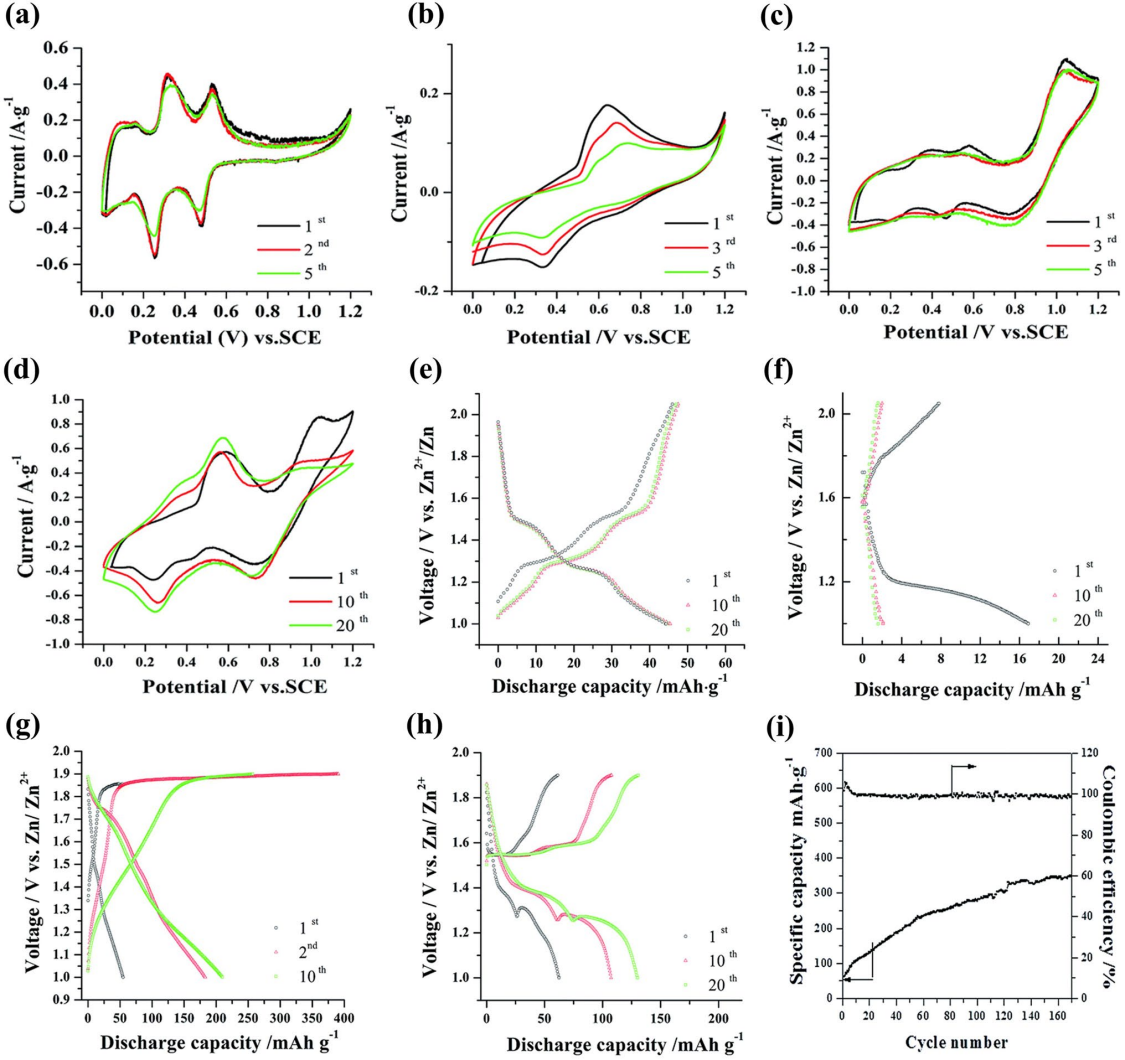
CV curves at a scan rate of 1 mV s^−1^ of Na_0.44_MnO_2_ in different electrolytes: **a** 1 M Na_2_SO_4_; **b** 1 M Na_2_SO_4_ + 0.5 M ZnSO_4_; **c** 1 M Na_2_SO_4_ + 0.05 M MnSO_4_; **d** 1 M Na_2_SO_4_ + 0.5 M ZnSO_4_ + 0.05 M MnSO_4_. Charge–discharge profiles of Na_0.44_MnO_2_ at 100 mA g^−1^ in different electrolytes: **e** 1 M Na_2_SO_4_; **f** 1 M Na_2_SO_4_ + 0.5 M ZnSO_4_; **g** 1 M Na_2_SO_4_ + 0.05 M MnSO_4_; **h** 1 M Na_2_SO_4_ + 0.5 M ZnSO_4_ + 0.05 M MnSO_4_.** i** Cycling performance of Na_0.44_MnO_2_ in 1 M Na_2_SO_4_ + 0.5 M ZnSO_4_ + 0.05 M MnSO_4_ at 100 mA g^−1^. Zinc sheets as counter electrodes [93]. Copyright 2016, Royal Society of Chemistry

**Fig. 10:**
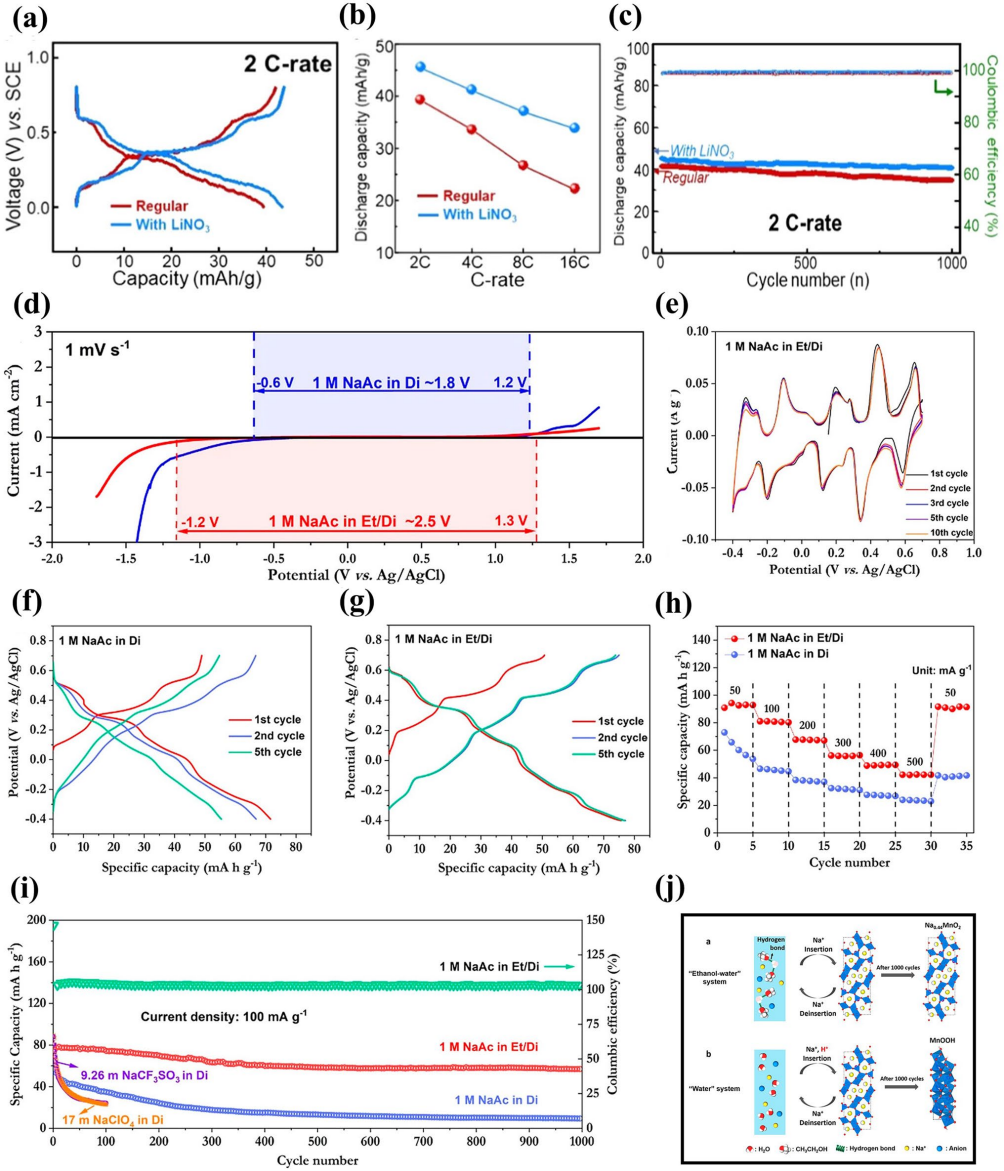
**a** Charge–discharge profiles, **b** Rate capabilities, **c** Cycling performance of Na_0.39_MnO_2_|AC full cells using 1 M NaClO_4_ electrolytes without and with LiNO_3_ (0.1 M) addition [94]. Copyright 2020, American Chemical Society. **d** Electrochemical stability of Na_0.44_MnO_2_ electrode in 1 M NaAc-Di and 1 M NaAc-Et/Di electrolytes. **e** CV profiles of Na_0.44_MnO_2_ electrode in 1 M NaAc–Et/Di electrolyte. Charge–discharge profiles of Na_0.44_MnO_2_ electrode in **f** 1 M NaAc-Di and **g** 1 M NaAc-Et/Di electrolytes. **h** Rate capability comparison and **i** Cycling performance of Na_0.44_MnO_2_ electrode in 1 M NaAc-Di and 1 M NaAc-Et/Di electrolytes. Using platinum foil and Ag/AgCl as counter and reference electrodes. **j** Schematics of ions storage in water and ethanol–water systems [96]. Copyright 2020, American Chemical Society

Second, electrolyte additives were adopted to improve the electrochemical performance of Na_*x*_MnO_2_ cathode. Bai et al. investigated the effect of addition of ZnSO_4_ and MnSO_4_ into Na_2_SO_4_ solution on electrochemical performance of Na_0.44_MnO_2_ cathode [93]. The Na_0.44_MnO_2_ electrode in various electrolytes exhibited different CV curves (Fig. 9a–d). With the addition of ZnSO_4_ and MnSO_4_, there were new redox couples observed, which could be related to the intercalation/deintercalation of Zn ions and oxidation/reduction of Mn^2+^. Therefore, the Na_0.44_MnO_2_ electrode in various electrolytes also displayed different charge–discharge behavior (Fig. 9e–h). With ZnSO_4_ addition, the discharge capacity decreased from 45 to 17 mAh g^− 1^, and became very small in the subsequent cycles (Fig. 9f). The rapid fading of capacity implied crystal structure change of Na_0.44_MnO_2_. It might be inferred that some Zn ions were inserted into Na_0.44_MnO_2_ resulting in collapse of crystal structure. In contrast, with MnSO_4_ addition, the discharge capacity was enhanced significantly (Fig. 9g), which might be caused by overcharge process. However, with the addition of ZnSO_4_ and MnSO_4_, the voltage plateaus became more significant and the discharge capacity increased (Fig. 9h), which could be attributed to synergistic effect between Mn and Zn ions in addition to quasi-reversible deposition/dissolution process of Mn ions. The discharge capacity of Na_0.44_MnO_2_ electrode in 1 M Na_2_SO_4_ + 0.5 M ZnSO_4_ + 0.05 M MnSO_4_ electrolyte increased dramatically and then remained steady with increasing cycling number (Fig. 9i). Thus, the addition of Zn and Mn ions in aqueous electrolytes has significantly influence on electrochemical performance of Na_0.44_MnO_2_ electrode. Except for ZnSO_4_ and MnSO_4_, LiNO_3_ was also used to improve the electrochemical performance of tunnel-type Na_0.39_MnO_2_ cathode [94]. A full cell Na_0.39_MnO_2_|AC with 1 M NaClO_4_ + 0.1 M LiNO_3_ solution delivered an increased discharge capacity of 45.1 mAh g^− 1^ at 1C (60 mA g^− 1^) (Fig. 10a), ultrafast rate capability with a capacity increase of 43% at 16C (Fig. 10b), and superior cycling stability with capacity retention increased from 84.1 to 90.3% after 1000 cycles (Fig. 10c), compared with a full cell without LiNO_3_ addition in electrolyte. The improved electrochemical performance with LiNO_3_ addition could be ascribed to increased ionic conductivity of electrolyte solution, co-intercalation of Na ions and Li ions, and lower surface resistance of cathode. Li ions established additional diffusion paths, which activated Na sites. In addition, Guo et al. reported an electrolyte additive of sodium dodecyl sulfate (SDS) for aqueous sodium/zinc battery [95]. The addition of SDS could form an artificial passivation film on Na_0.44_MnO_2_ electrode. The passivation film could reduce the formation of the insulating by-product Zn_4_SO_4_(OH)_6_·xH_2_O on Na_0.44_MnO_2_ surface and inhibit the dissolution of Na_0.44_MnO_2_. Therefore, a Na_0.44_MnO_2_|Zn battery using SDS-modified aqueous electrolyte displayed excellent cycling stability with capacity retention of 93% after 1500 cycles compared with the battery without SDS addition (only 45% capacity retention).

**Fig. 11:**
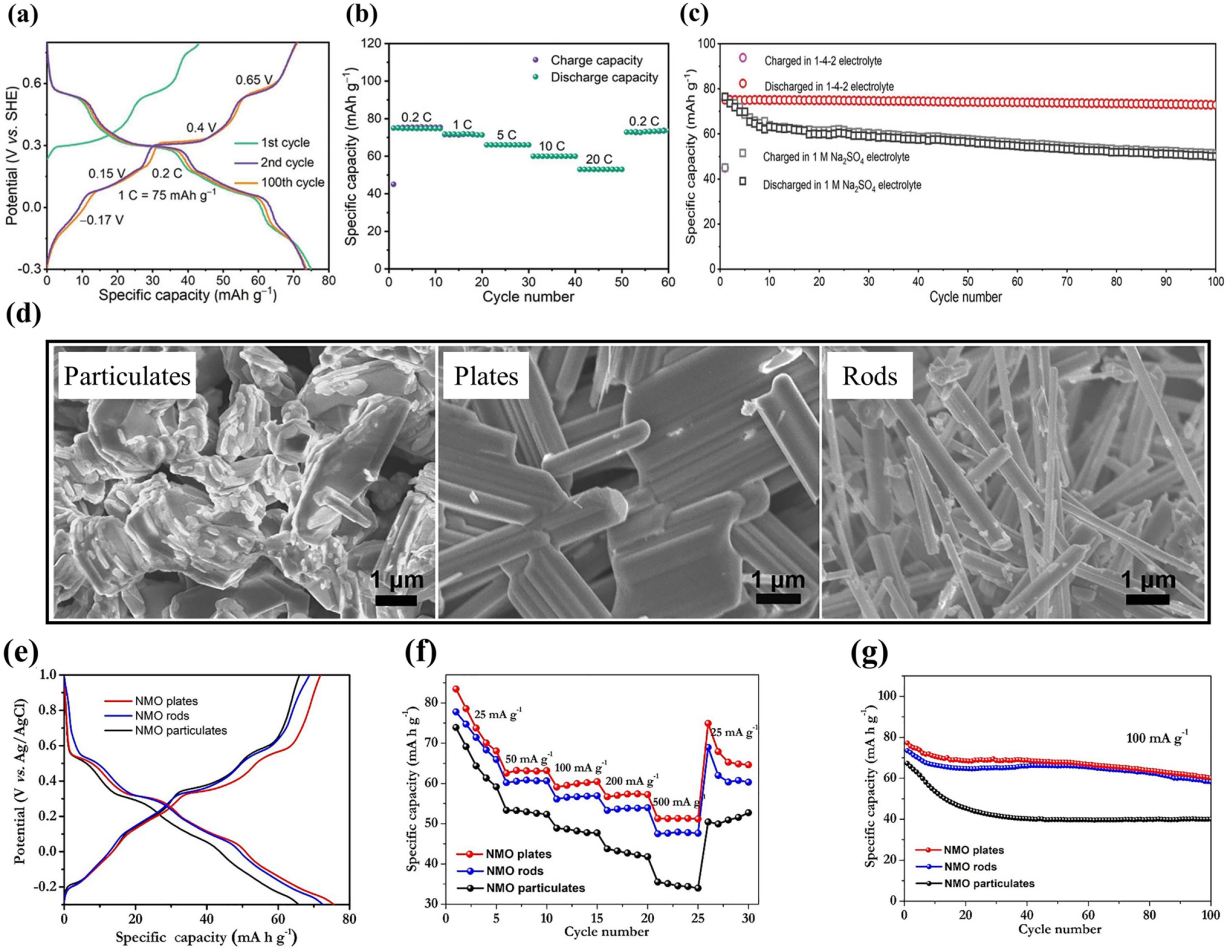
**a** Charge–discharge curves and **b** Rate performance of Na_0.44_MnO_2_ electrode in 1-4-2 electrolyte. **c** Cycling performance of Na_0.44_MnO_2_ electrode in 1-4-2 electrolyte and in 1 M Na_2_SO4 electrolyte [97]. Copyright 2022, Wiley‐VCH. **d** SEM images of the synthesized Na_0.44_MnO_2_ samples. **e** Charge and discharge curves from the second cycle of the Na_0.44_MnO_2_ samples with different shapes at 100 mA g^− 1^.** f** Rate capability of the Na_0.44_MnO_2_ samples with different shapes. **g** Cycling performance of the Na_0.44_MnO_2_ samples with different shapes at 100 mA g^− 1^ [98]. Copyright 2019, Elsevier

Third, solvent was optimized to improve the electrochemical performance of Na_*x*_MnO_2_ cathode. Chua et al. adopted hybrid electrolytes with an ethanol-rich media to attain highly stable Na-ion electrochemistry [96]. An ethanol–water solvent with ethanol–water (Et-Di) volume ratio of 5:1 was used due to the lowest contact angle, and hydrogen bonds were readily formed between ethanol and water molecules. In 1 M NaAc (sodium acetate)-Et/Di electrolyte, a wider electrochemical window of ~ 2.5 V was obtained (Fig. 10d), and the Na_0.44_MnO_2_ electrode displayed overlapping CV curves (Fig. 10e), indicating highly reversible insertion/extraction process of Na ions. The discharge capacities were 71.6 and 76.8 mAh g^− 1^ at 100 mA g^− 1^ in 1 M NaAc-Di and 1 M NaAc-Et/Di electrolytes (Fig. 10f-g), respectively. Notably, the Na_0.44_MnO_2_ electrode in 1 M NaAc-Et/Di electrolyte demonstrated much better rate capability (Fig. 10h) and excellent cycling stability (Fig. 10i). The improved electrochemical performance might be attributed to the intrinsic hydrogen-bonding interaction suppressing the water proton’s activity. Figure 10j exhibits schematics of the structural evolution and ions storage of Na_0.44_MnO_2_ electrode in NaAc-Di and NaAc-Et/Di systems. In water system, Na ions and protons could co-insert into Na_0.44_MnO_2_ electrode, and Na_0.44_MnO_2_ suffered Mn^2+^ dissolution and irreversible phase transformation to MnOOH during cycling process. In contrast, water proton activity was effectively suppressed by hydrogen bonds with ethanol oxygens in the ethanol–water system. Therefore, the ethanol–water system could result in much higher electrochemical performance. In addition, a deep eutectic electrolyte was developed to improve the electrochemical performance of Na_0.44_MnO_2_ cathode by Hou et al. [97]. The deep eutectic electrolyte consisted of 1 mol NaClO_4_·H_2_O, 3 mol water and 2 mol urea (named 1-4-2 electrolyte), which had low eutectic point of − 19 °C. The Na_0.44_MnO_2_ electrode in the 1-4-2 electrolyte with Pt as counter electrode exhibited a discharge capacity of 75.16 mAh g^− 1^ at 0.2C (Fig. 11a) and outstanding rate capacity with discharge capacity of 53.29 mAh g^− 1^ at 20C (Fig. 11b). More importantly, the Na_0.44_MnO_2_ electrode in the 1-4-2 electrolyte demonstrated a longer cycle life with capacity retention of 95% compared with the Na_0.44_MnO_2_ electrode in 1 M Na_2_SO_4_ electrolyte (65% capacity retention) (Fig. 11c). The water activity and Mn dissolution were suppressed in the 1-4-2 eutectic electrolyte, which were helpful for maintaining the structural integrity of Na_0.44_MnO_2_ and improving the cycling stability. Thus, optimizing the solvent can effectively enhance the electrochemical performance.

In a word, the electrochemical performance of Na_*x*_MnO_2_ electrodes could be effectively improved by optimizing electrolyte salt concentration, choosing suitable electrolyte additive and optimizing solvent. Therefore, optimizing electrolyte is a very effective method for improving electrochemical performance of aqueous batteries.

(2) **Optimization of Morphology**

Morphology of electrode materials affects their electrochemical performance. Chua et al. synthesized Na_0.44_MnO_2_ rods and Na_0.44_MnO_2_ plates by sol–gel method and Na_0.44_MnO_2_ particulates by solid-state method, and investigated their electrochemical performance in 1 M Na_2_SO_4_ solution using Pt foil as counter electrode [98]. The SEM images of the synthesized Na_0.44_MnO_2_ samples were shown in Fig. 11d. Among the three samples, Na_0.44_MnO_2_ plates delivered the highest discharge capacity of 77.2 mAh g^− 1^ at 100 mA g^− 1^, as shown in Fig. 11e. Compared with Na_0.44_MnO_2_ particulates and rods, Na_0.44_MnO_2_ plates exhibited superior rate capability, and delivered discharge capacities of 83.5, 59.1 and 51.3 mAh g^− 1^ at 25, 100 and 500 mA g^− 1^ (Fig. 11f). In addition, the Na_0.44_MnO_2_ plates also showed excellent cycling performance with a capacity of about 60 mAh g^− 1^ after 100 cycles (Fig. 11g). The excellent electrochemical performance of Na_0.44_MnO_2_ plates could be ascribed to the chemical bonded plate structure and the formation of sheet-like Na-birnessite layer on the surface of Na_0.44_MnO_2_ plates during charge–discharge cycling. Therefore, the electrochemical performance of Na_*x*_MnO_2_ cathode materials can be improved by optimizing their morphology.

(3) **Element Doping or Substitution**

**Fig. 12:**
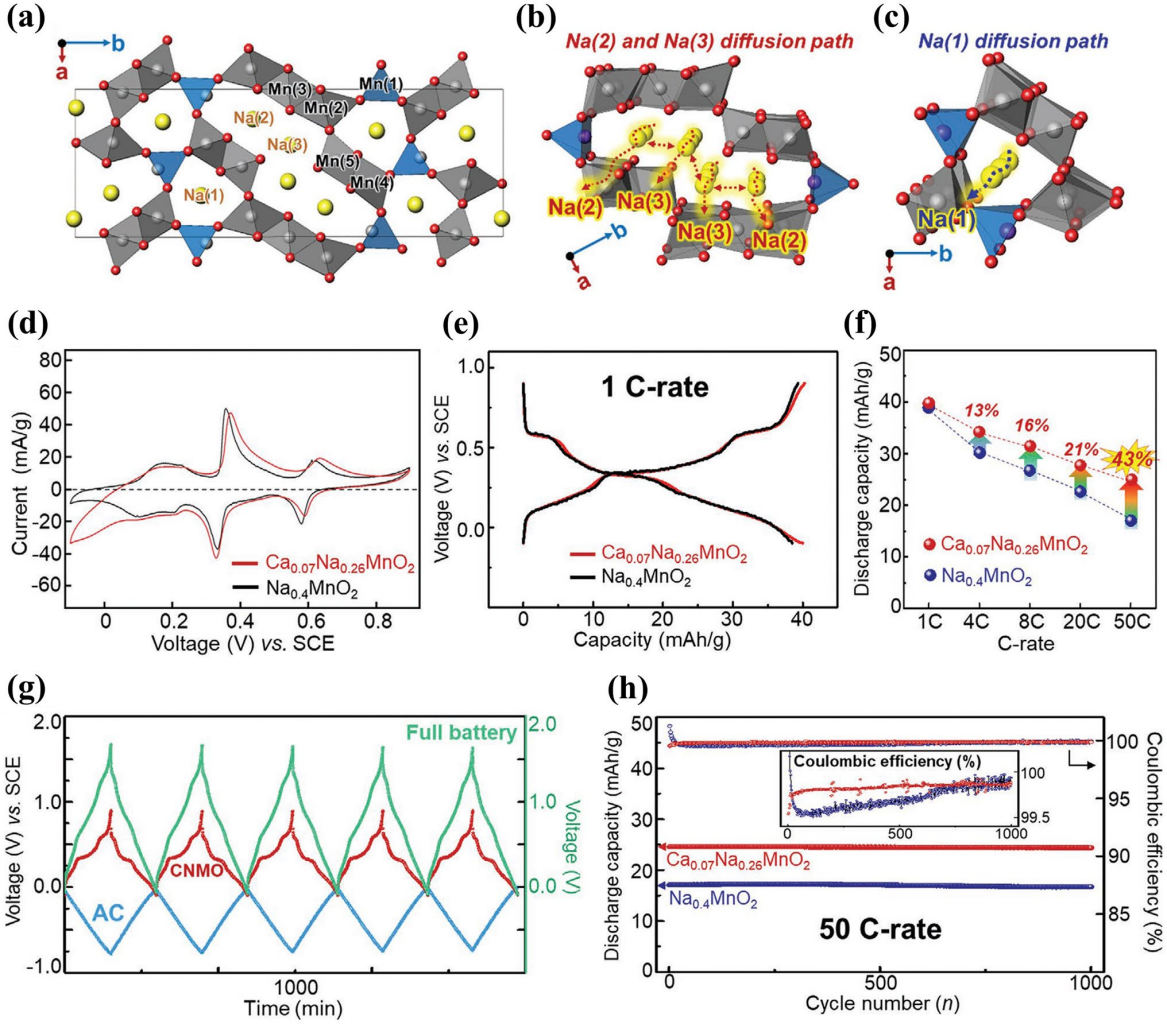
**a** Unit cell crystal structure, **b** Na(2) and Na(3) diffusion pathways and **c** Na(1) diffusion pathway of tunnel-type Na_0.4_MnO_2_. **d** CV curves at a scan rate of 0.1 mV s^− 1^ and **e** Charge/discharge curves at 1C of Na_0.4_MnO_2_ and Ca_0.07_Na_0.26_MnO_2_ electrodes in 1 M NaClO_4_ aqueous solution. **f** Rate capability, **g** Charge–discharge profiles, and **h** Cycling performance at 50C of a Ca_0.07_Na_0.26_MnO_2_|AC full cell with 1 M NaClO_4_ aqueous solution electrolyte [45]. Copyright 2020, Wiley‐VCH

Element doping is an effective way to improve the electrochemical properties of electrode materials. Calcium-doped Na_0.4_MnO_2_, Ca_0.07_Na_0.26_MnO_2_, as cathode material was investigated in 1 M NaClO_4_ solution by Chae et al. [45]. Figure 12a-c shows the crystal structure of tunnel-type Na_0.4_MnO_2_ and its local diffusion pathways of Na(1), Na(2) and Na(3) sites. The doped calcium was placed at the Na(1) site, reduced the neighboring manganese, and formed sodium defects (Na(2) and Na(3) sites) inside the manganese oxide framework. Compared with Na_0.4_MnO_2_, Ca_0.07_Na_0.26_MnO_2_ exhibited a broad CV curve with three redox peaks (Fig. 12d). The charge–discharge curve of Ca_0.07_Na_0.26_MnO_2_ electrode also showed three voltage regions divided clearly and a discharge capacity of 40 mAh g^− 1^ at 1C (Fig. 12e). The Ca_0.07_Na_0.26_MnO_2_ electrode demonstrated much enhanced rate capability compared with the Na_0.4_MnO_2_ electrode with an increase of 43% at 50C (Fig. 12f). A Ca_0.07_Na_0.26_MnO_2_|AC full cell displayed stable operation of both anode and cathode sides (Fig. 12g) and exhibited superior cycling stability with capacity retention of 98.8% after 1000 cycles (Fig. 12h). The cycling stability could be attributed to the presence of calcium cations (Ca^2+^) in the structure. Therefore, the calcium doping improved the rate capability and cycling stability of Na_0.4_MnO_2_ electrode.

Partial substitution for Mn in Na_*x*_MnO_2_ is another effective approach to improve its electrochemical performance. Ti-substituted Na_x_Mn_x_Ti_1-x_O_2_ cathode materials were investigated for aqueous SIBs. Wang et al. reported a Na-rich tunnel-type Na_0.66_Mn_0.66_Ti_0.34_O_2_ cathode material [99]. A full cell Na_0.66_Mn_0.66_Ti_0.34_O_2_|NaTi_2_(PO_4_)_3_/C with 1 M Na_2_SO_4_ electrolyte showed a higher reversible capacity of 76 mAh g^− 1^ at 236 mA g^− 1^ (2C), which was much higher than that of Na_0.44_Mn_0.44_Ti_0.56_O_2_|NaTi_2_(PO_4_)_3_/C full cell (45 mAh g^− 1^). In addition, the Na_0.66_Mn_0.66_Ti_0.34_O_2_|NaTi_2_(PO_4_)_3_/C full cell also displayed excellent rate performance with a discharge capacity of 54 mAh g^− 1^ at 10C and long-term cycling stability with capacity retention of 89% after 300 cycles at 2C. With increasing Ti content, a Na_0.5_Mn_0.5_Ti_0.5_O_2_ electrode in 6 M NaClO_4_ aqueous electrolyte exhibited a discharge capacity of 46 mAh g^− 1^ at 30 mA g^− 1^ [100], and a Na_4_Mn_4_Ti_5_O_18_ (or Na_0.44_Mn_0.44_Ti_0.56_O_2_) electrode in 1 M Na_2_SO_4_ solution delivered an initial discharge capacity of 36 mAh g^− 1^ [101]. It can be found that the Ti substitution for Mn can improve the electrochemical performance, but increasing Ti content decreases the reversible discharge capacity. Apart from Ti substitution, partial substitutions of Cu, Fe, Ni and Co have also been reported. Boyd et al. investigated some P2 oxides in 1 M Na_2_SO_4_ aqueous solution, including Na_0.64_Mn_0.62_Cu_0.31_O_2_ (NaMCu), Na_0.64_Ni_0.22_Mn_0.66_Cu_0.11_O_2_ (NaNMCu), Na_0.62_Ni_0.22_Mn_0.66_Fe_0.10_O_2_ (NaNMFe) and Na_0.61_Ni_0.22_Mn_0.66_Co_0.10_O_2_ (NaNMCo) [102]. These oxides were synthesized by coprecipitation method and had similar structures and morphologies. The anodic capacities in the first cycle were 30.4, 32.0, 47.7 and 60.9 mAh g^− 1^ for NaMCu, NaNMCu, NaNMFe and NaNMCo, respectively. Although NaNMCo displayed higher initial anodic capacity, the water intercalation and phase transformation resulted in microscopic exfoliation and severe damage occurred in NaNMCo. In addition, a Na_0.8_Ni_0.33_Co_0.33_Mn_0.33_O_2_ cathode material was reported by Nwanya et al. [103]. The synthesized powders consisted of sheath-like nanoparticles and quasi-spherical nanoparticles. The Na_0.8_Ni_0.33_Co_0.33_Mn_0.33_O_2_ electrode showed a discharge capacity of 86 mAh g^− 1^ at 50 mA g^− 1^ using Pt as counter electrode in 0.5 M Na_2_SO_4_ solution. Therefore, element doping and partial substitution for Mn in Na_*x*_MnO_2_ can greatly enhance its electrochemical performance.

(4) **Carbon Modification**

**Fig. 13:**
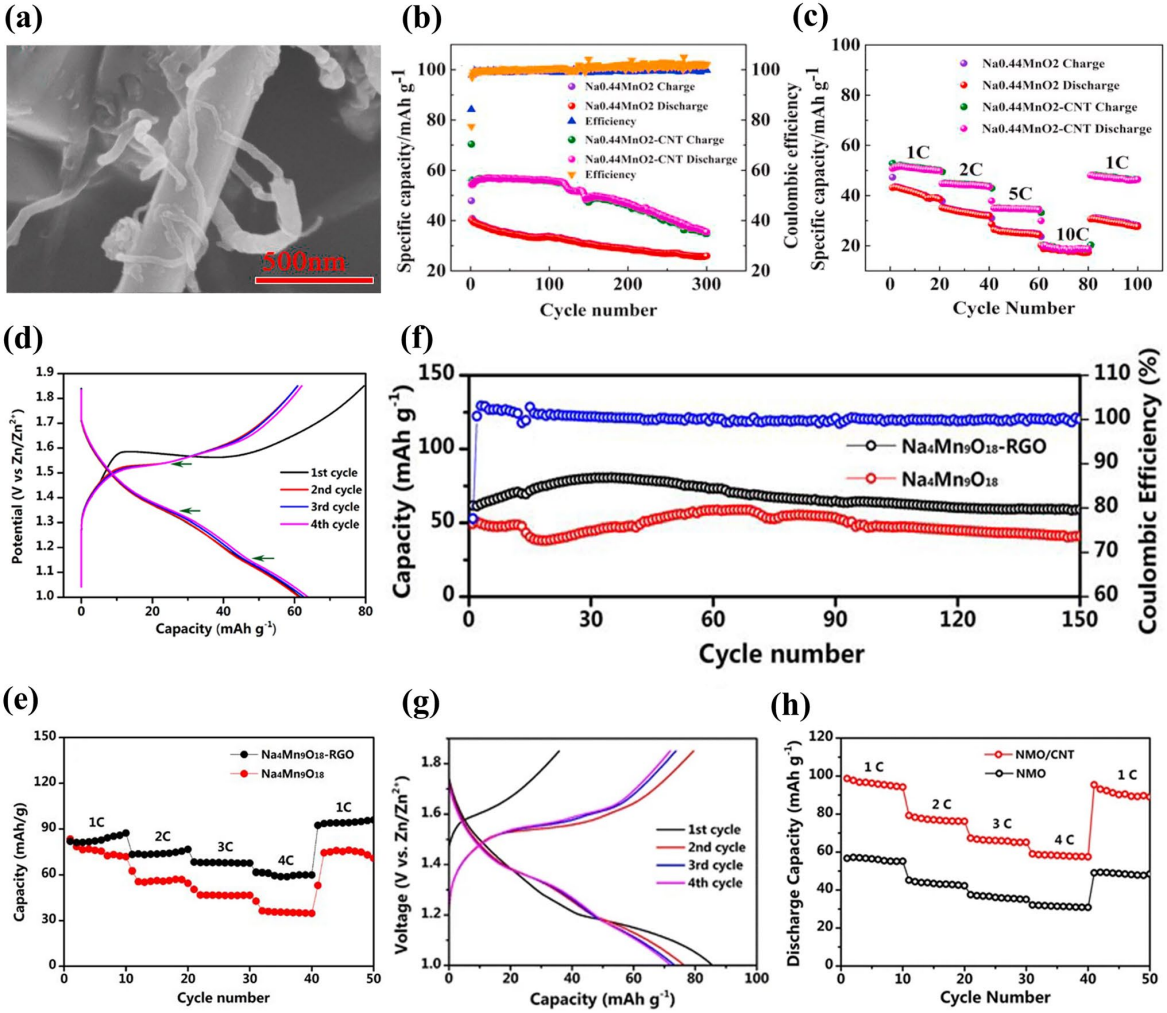
**a** SEM image of the synthesized Na_0.44_MnO_2_-CNT.** b** Cycling performance at 1C and **c** Rate performance of Na_0.44_MnO_2_-CNT and Na_0.44_MnO_2_ electrodes in 1 M Na_2_SO_4_ solution with Pt counter electrode [104]. Copyright 2020, Elsevier.** d** Charge/discharge curves at 4C of Na_4_Mn_9_O_18_-RGO electrode in 1 M Na_2_SO_4_ + 0.5 M ZnSO_4_ solution using Zn as counter electrode. **e** Cycling performance of Na_4_Mn_9_O_18_ and Na_4_Mn_9_O_18_-RGO electrodes at 4C. **f** Rate capability of Na_4_Mn_9_O_18_ and Na_4_Mn_9_O_18_-RGO electrodes. [105]. Copyright 2017, The authors. **g** Discharge/charge profiles at 4C of Na_4_Mn_9_O_18_/CNT electrode in 1 M Na_2_SO_4_ + 0.5 M ZnSO_4_ solution using Zn as counter electrode.** h** Rate performance of Na_4_Mn_9_O_18_/CNT (NMO/CNT) and Na_4_Mn_9_O_18_ (NMO) electrodes [110]. Copyright 2017, The Author(s)

Carbon nanotubes (CNT) and reduced graphene oxide (RGO) has been used to improve the electrochemical performance of electrode materials. Gu et al. investigated the effect of CNT wrapping on the electrochemical performance of Na_0.44_MnO_2_ [104]. CNT wrapping rod-like Na_0.44_MnO_2_ (Na_0.44_MnO_2_-CNT) was synthesized by solid-state method, and its morphology is shown in Fig. 13a. The CNT wrapping around Na_0.44_MnO_2_ particles enhanced the electronic conductivity of Na_0.44_MnO_2_. The Na_0.44_MnO_2_-CNT electrode in 1 M Na_2_SO_4_ solution demonstrated a charge capacity of 70.4 mAh g^− 1^ at 1C (50 mA g^− 1^), which was higher than that of Na_0.44_MnO_2_ electrode (Fig. 13b). After 300 cycles, the reversible capacity retention was about 63.4% for Na_0.44_MnO_2_-CNT electrode. The Na_0.44_MnO_2_-CNT electrode also showed better rate performance compared with Na_0.44_MnO_2_ electrode (Fig. 13c). Therefore, CNT wrapping could improve reversible capacity, rate capability and cycling performance of Na_0.44_MnO_2_-CNT electrode.

In addition, Na_4_Mn_9_O_18_ (more commonly Na_0.44_MnO_2_) materials were modified by RGO or CNT for enhancing their electrochemical performance. Yin et al. prepared spherical Na_4_Mn_9_O_18_-RGO composites by spray-drying method [105]. The Na_4_Mn_9_O_18_-RGO electrode in 1 M Na_2_SO_4_ + 0.5 M ZnSO_4_ solution showed a discharge capacity of 61.7 mAh g^− 1^ at 4C in the first cycle (Fig. 13d), which was higher than that of reported Na_4_Mn_9_O_18_ electrodes [106–108]. The Na_4_Mn_9_O_18_-RGO electrode also displayed higher rate capability and good cycle stability compared with Na_4_Mn_9_O_18_ electrode (Fig. 13e-f). The addition of RGO could form interlaced network, which provided fast electron conduction pathways and held the mechanical stresses induced by insertion/extraction of Na ions. Thus, a free-standing Na_4_Mn_9_O_18_-RGO composite film also delivered a high discharge capacity of 83 mAh g^− 1^ at 100 mA g^− 1^ in 0.5 M NaCH_3_COO + 0.5 M Zn(CH_3_COO)_2_ solution using Zn counter electrode [109]. Apart from Na_4_Mn_9_O_18_-RGO composites, Yin et al. also prepared Na_4_Mn_9_O_18_/CNT composites with microspherical structure by spray-drying method [110]. The Na_4_Mn_9_O_18_/CNT electrode in 1 M Na_2_SO_4_ + 0.5 M ZnSO_4_ solution showed an initial discharge capacity of 85.6 mAh g^− 1^ at 4 C (Fig. 13g), which was higher than that of Na_4_Mn_9_O_18_-RGO electrode [105]. Compared with the Na_4_Mn_9_O_18_ electrode, the Na_4_Mn_9_O_18_/CNT electrode also exhibited good rate capability (Fig. 13h) and cycling stability. The superior electrochemical performance of the Na_4_Mn_9_O_18_/CNT electrode could be ascribed to spherical structure and CNT addition, which improved the conductivity of the composites. Based on the advantages of CNT and RGO, Shan et al. prepared Na_4_Mn_9_O_18_/CNT/RGO composites with microsphere structure [111]. Compared with Na_4_Mn_9_O_18_, Na_4_Mn_9_O_18_/CNT and Na_4_Mn_9_O_18_/RGO electrodes, the Na_4_Mn_9_O_18_/CNT/RGO electrode in 0.5 M Na_2_SO_4_ + 1 M ZnSO_4_ solution displayed a higher initial discharge capacity of 96.2 mAh g^− 1^ at 4C. Therefore, the modification of CNT and RGO can enhance the electronic conductivity of Na_*x*_MnO_2_ cathode materials, and improve effectively their reversible capacity, rate capability and cycling performance.

#### Others

Some other Mn-based oxides as cathode materials have also been reported for aqueous SIBs. Shan et al. synthesized a layered Mn_5_O_8_ material which had a well-ordered hydroxylated interphase, and investigated its electrochemical performance [112]. A symmetric full cell with Mn_5_O_8_ electrodes (1 M Na_2_SO_4_ electrolyte) showed nearly linear potential-capacity curves at various current densities and characteristic of pseudocapacitive response, and delivered a discharge capacity of 116 mAh g^− 1^ at 5000 mA g^− 1^ and good cycling stability. This system suppressed the oxygen and hydrogen evolution reactions, exhibited a high-stable potential window of 3.0 V, and demonstrated a two-electron charge transfer reaction involving Mn^2+^/Mn^4+^ redox couple by means of the interplay between the unique bivalence structure and hydroxylated interphase of Mn_5_O_8_. Subsequently, Shan et al. also synthesized high purity Mn_5_O_8_ nanoparticles by oxidation of Mn_3_O_4_ spinel, and the synthesized nanoparticles were binary Mn_5_O_8_ expressed as [Mn^2+^_2_][Mn^4+^_3_O^2−^_8_] [113]. A symmetric full cell constructed using Mn_5_O_8_ electrodes and 1 M Na_2_SO_4_ solution displayed a stable discharge capacity of about 103 mAh g^− 1^ at 5000 mA g^− 1^, and excellent cycling performance without capacity fade upon 5000 cycles.

In conclusion, Mn-based oxides, such as MnO_2_, Mn_5_O_8_ and Na_*x*_MnO_2_, have been extensively investigated as cathode materials for aqueous SIBs. Different Mn-based oxides showed various electrochemical performance, and their electrochemical performance can be improved by electrolyte optimization, morphology optimization, element doping or substitution, and carbon modification. A comprehensive summary of the electrochemical performance of some Mn-based oxides discussed previously is presented in Table 1.

### Prussian Blue Analogues

Prussian blue analogues have been widely investigated as cathode materials [14, 114, 115]. Open-framework structures of Prussian blue analogues possess wide channels, which allow rapid insertion/extraction of Na ions in aqueous solution. In this part, some Mn-based Prussian blue analogues will be introduced, including their electrochemical performance and improvement methods.

#### Electrochemical Performance

**Fig. 14:**
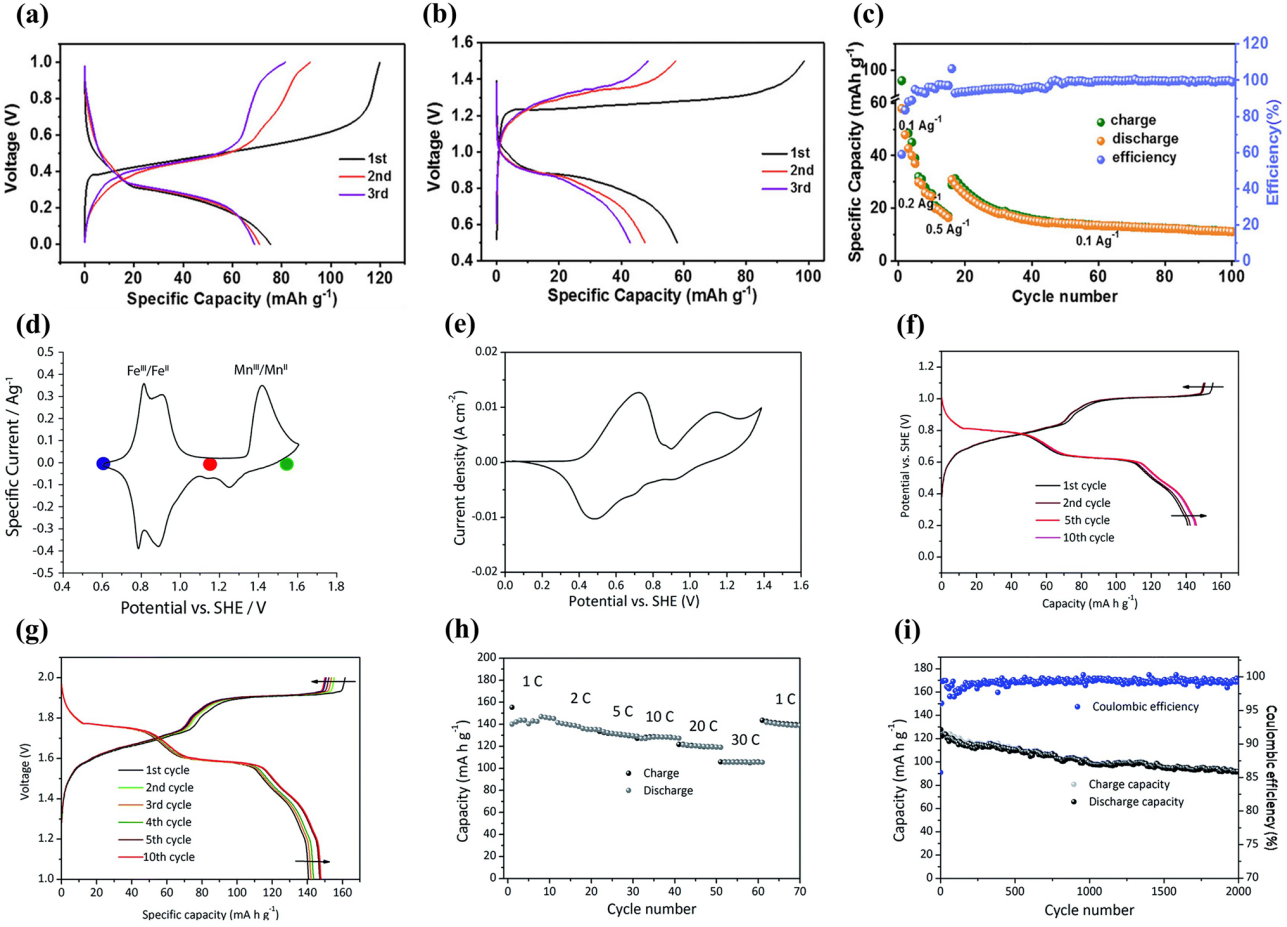
**a** Charge–discharge curves of Na_2_MnFe(CN)_6_ electrode at 100 mA g^− 1^ in WiSE electrolyte using AC as counter electrode. **b** Charge–discharge curves at 100 mA g^− 1^ and **c** Rate performance of Na_2_MnFe(CN)_6_|WiSE|NaTi_2_(PO_4_)_3_/C full cell [116]. Copyright 2018, Wiley‐VCH. **d** CV curve of MnHCFe in a saturated NaClO_4_ solution (pH = 2) using AC anode [121]. Copyright 2016, Royal Society of Chemistry **e** CV curve of Na_2_MnFe(CN)_6_ electrode in aqueous electrolyte (1 M Na_2_SO_4_ + 1 M ZnSO_4_) with SDS addition at 5 mV s^− 1^ (standing for one day before testing). **f** Charge/discharge profiles of Na_2_MnFe(CN)_6_ electrode in aqueous electrolyte (1 M Na_2_SO_4_ + 1 M ZnSO_4_) with SDS addition at 0.5C. **g** Charge/discharge profiles at 0.5C, **h** Rate capability and **i** Cycling performance at 5C of Na_2_MnFe(CN)_6_|Zn full cell using the electrolyte with SDS addition [22]. Copyright 2017, Royal Society of Chemistry

Sodium manganese hexacyanoferrate, Na_x_MnFe(CN)_6_, is a promising cathode material for aqueous SIBs. Na_2_MnFe(CN)_6_ material was prepared by precipitation method and its electrochemical performance was studied using NaTi_2_(PO_4_)_3_ as anode in 1 M Na_2_SO_4_ aqueous solution [42]. The Na_2_MnFe(CN)_6_ electrode delivered a reversible discharge capacity of about 85 mAh g^− 1^ at 1C, but the discharge capacity decreased to 66.8 mAh g^− 1^ after 30 cycles, indicating poor cycling stability. In a “water-in-salt” electrolyte (WiSE) with sodium acetate (8 M) + potassium acetate (32 M), the Na_2_MnFe(CN)_6_ electrode displayed a discharge capacity of 75 mAh g^− 1^ at 100 mA g^− 1^ using AC as counter electrode (Fig. 14a) [116]. However, a Na_2_MnFe(CN)_6_|WiSE|NaTi_2_(PO_4_)_3_/C full cell showed a discharge capacity of only 57 mAh g^− 1^ at 100 mA g^− 1^ (Fig. 14b) and poor rate performance (Fig. 14c), due to irreversible deintercalation of Na ions from NaTi_2_(PO_4_)_3_/C and the instability of Na_2_MnFe(CN)_6_ in the alkaline environment. The WiSE consisting of sodium acetate and potassium acetate was a hybrid electrolyte, and the battery based on the WiSE could be called Na–K hybrid battery, where Na^+^/K^+^ hybrid-ion electrolyte could enhance electrochemical performances of battery [117]. Moreover, in 17 M NaClO_4_ aqueous electrolyte, a Na_2_MnFe(CN)_6_|Na_3_Fe_2_(PO_4_)_3_ full cell presented a discharge capacity of 31 mAh g mAh g^− 1^ (based on the mass of cathode and anode) and energy density of 27 Wh kg^− 1^ [118]. The full cell exhibited promising rate performance and good cycling performance with 75% capacity retention after 700 cycles. In contrast, when using K_0.01_Cr_3_[Cr(CN)_6_]_2_·3.8H_2_O as anode, a Na_2_MnFe(CN)_6_|K_0.01_Cr_3_[Cr(CN)_6_]_2_·3.8H_2_O full cell displayed a high discharge capacity of 52.8 mAh g^− 1^ at 1C (based on the mass of cathode and anode), corresponding to a high energy density of 81.6 Wh kg^− 1^ [119]. The full cell also displayed excellent rate performance with a discharge capacity of 23 mAh g^− 1^ at 150C and excellent cycling stability with capacity retention of 93% after 500 cycles at 30C. These researches suggest that aqueous electrolyte and counter electrode have a great influence on the electrochemical performance of Na_2_MnFe(CN)_6_ electrodes. Accordingly, by using graphite/amorphous carbon film as the current collector, a prototype pouch cell stacking using six Na_2_MnFe(CN)_6_|NaTi_2_(PO_4_)_3_ bipolar electrodes and “water-in polymer” gel electrolyte delivered a discharge capacity of 114 mAh g^− 1^ at 1C and had an energy density of 86 Wh kg^− 1^ at 23 W kg^− 1^ (based on the mass of cathode and anode) [120]. The prototype pouch cell also demonstrated excellent rate capability with a discharge capacity of 86 mAh g^− 1^ at 30C and pre-long cycling performance with 80% capacity maintained after 4000 cycles at 10C.

Apart from Na_2_MnFe(CN)_6_, Na_1.33_Mn[Fe(CN)_6_]_0.79_·γ_0.21_·1.88H_2_O (γ = Fe(CN)_6_ vacancy) (NaMnHCFe) was also investigated as cathode material [121]. Figure 14d shows the CV curve of NaMnHCFe electrode in a saturated (10 M) NaClO_4_ solution using AC anode. Three electrochemical processes could be observed at 0.8, 0.9 and 1.4 V, which were ascribed to the electrochemical activity of C-coordinated Fe and N-coordinated Mn. The NaMnHCFe electrode showed a high discharge capacity of 125 mAh g^− 1^ at 1C (120 mA g^− 1^) with a low coulombic efficiency of 83%. The reversible capacity of NaMnHCFe was very high; however, there was no report on its rate and cycling performance.

#### Improvement Methods

Although Mn-based Prussian blue analogues displayed high reversible capacity, they usually showed low rate capability and poor cycle stability due to poor conductivity and easy collapse of structure. Some improvement methods have been developed to improve the electrochemical performance of Mn-based Prussian blue analogues, including electrolyte optimization, carbon modification, and optimization of vacancies in Mn-based Prussian blue analogues.

1. **Optimization of Electrolyte**

First, electrolyte additives were adopted to enhance the electrochemical performance of batteries. Hou et al. investigated the effect of SDS addition to aqueous electrolyte (1 M Na_2_SO_4_ + 1 M ZnSO_4_) on electrochemical performance of a hybrid battery using Na_2_MnFe(CN)_6_ nanocubes as cathode and Zn as anode [22]. The addition of SDS expanded the electrochemical stability window to about 2.5 V, and there were two cathodic peaks (0.7 and 1.1 V) and three reduction peaks (0.4, 0.7 and 0.9 V) in the CV curve (Fig. 14e). The Na_2_MnFe(CN)_6_ electrode delivered a discharge capacity of 140 mAh g^− 1^ at 0.5C (80 mA g^− 1^) (Fig. 14f), which was higher than that of reported Na_2_MnFe(CN)_6_ [42]. Based on the electrolyte with SDS addition, a Na_2_MnFe(CN)_6_|Zn full cell displayed a discharge capacity of 137 mAh g^− 1^ at 0.5C (Fig. 14g) and a high energy density of about 170 Wh kg^− 1^ at 64 W kg^− 1^. The full cell also showed a high rate performance with a discharge capacity of 100 mAh g^− 1^ at 30C (Fig. 14h), which could be attributed to fast intercalation kinetics of Na_2_MnFe(CN)_6_ electrode. In particular, the full cell exhibited excellent cycling stability and the capacity retention after 2000 cycles was about 75% at 5C with coulombic efficiency of nearly 100% (Fig. 14i). The addition of SDS could effectively inhibit the water decomposition (evolution of oxygen or hydrogen), suppress the Mn dissolution and Zn corrosion, and improve the rate capability and cycle life.

Second, optimizing electrolyte salt concentration was used to improve the electrochemical performance of batteries. Nakamoto et al. reported the effect of NaClO_4_ concentration on electrochemical performance of Na_2_MnFe(CN)_6_ cathode [122]. The electrochemical window for 17 M NaClO_4_ aqueous electrolyte was 2.8 V, which was wider than that for 1 M NaClO_4_ (only 1.9 V). The discharge capacities of Na_2_MnFe(CN)_6_ electrodes in 1, 7, 14 and 17 M NaClO_4_ aqueous electrolytes were about 64, 119, 120 and 123 mAh g^− 1^ at 2.0 mA cm^−2^, respectively. Higher discharge capacity could be obtained in the electrolyte with higher salt concentration. With increasing the NaClO_4_ concentration, the cycle performance was also improved and the best cycling performance was achieved in highly concentrated (17 M) electrolyte. A Na_2_MnFe(CN)_6_|NaTi_2_(PO_4_)_3_ full cell with 17 M NaClO_4_ aqueous electrolyte exhibited a first discharge capacity of 117 mAh g^− 1^ at 2.0 mA cm^−2^ and capacity retention of 81% after 50 cycles. The high salt concentration could suppress water activity, decrease the amount of free water, and improve the electrochemical performance of aqueous batteries.

**Fig. 15:**
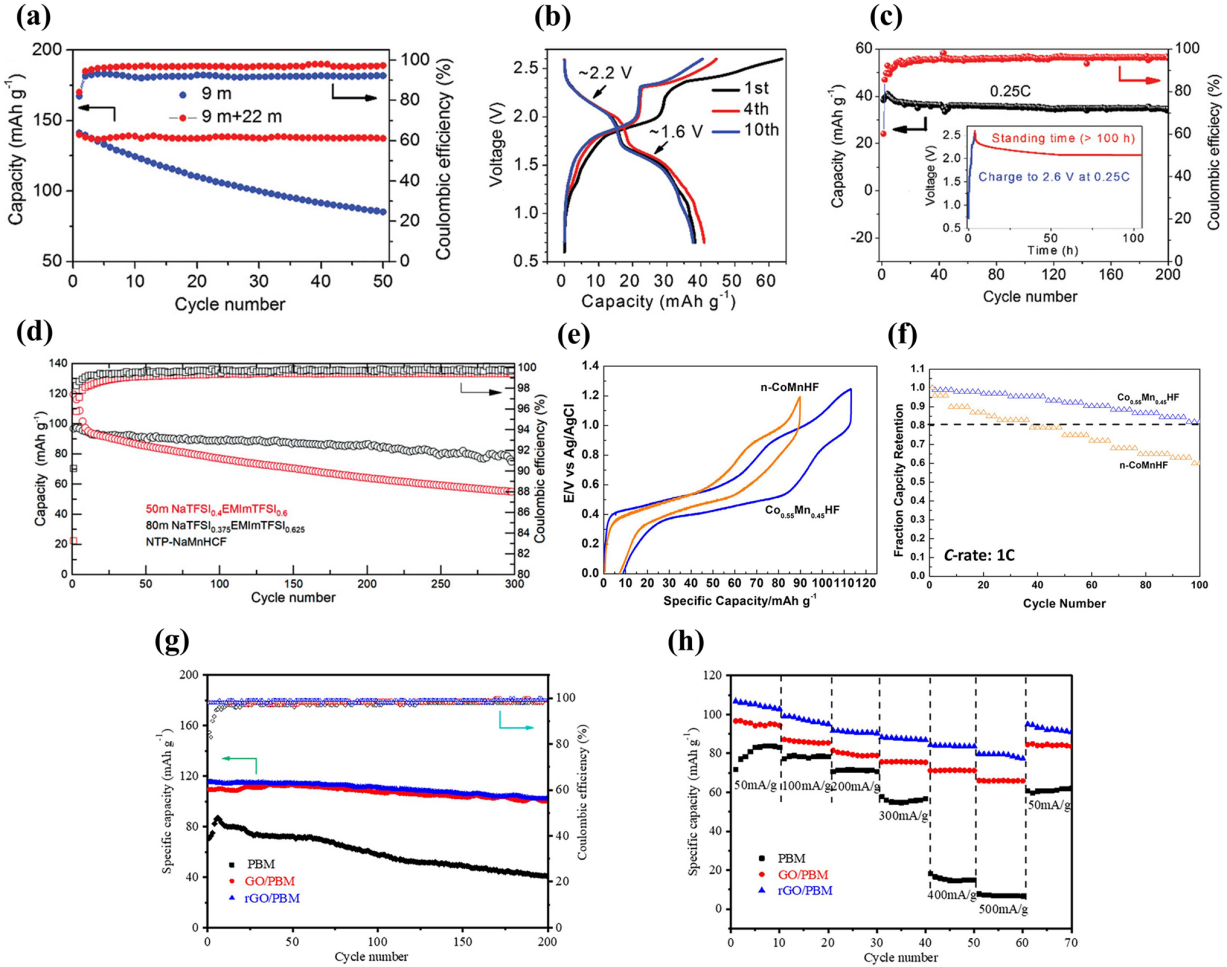
**a** Cycling performance of NaMnHCF electrode at 1C in 9 M NaOTF electrolyte and 9 M NaOTF + 22 M TEAOTF electrolyte. **b** Charge/discharge curves and **c** cycling performance of NaMnHCF|IC-WiS|NaTiOPO_4_ full battery at 0.25C (1C = 140 mA g^− 1^) [123]. Copyright 2019, Wiley‐VCH. **d** Cycling performance of Na_2_Mn[Fe(CN)_6_]|NaTi_2_(PO_4_)_3_ full cells with 80 M NaTFSI_0.375_EMImTFSI_0.625_ and 50 M NaTFSI_0.4_EMImTFSI_0.6_ electrolyte at 1C [124]. Copyright 2020, Wiley‐VCH. **e** Charge/discharge profiles and **f** Fraction capacity retention of Co_0.55_Mn_0.45_HF and *n*-CoMnHF electrodes at 1C in 1 M NaNO_3_ solution [125]. Copyright 2020, American Chemical Society.** g** Cycling performance at 85 mA g^− 1^ and** h** rate capabilities of PBM, GO/PBM, and RGO/PBM electrodes in 1 M Na_2_SO_4_ + 1 M ZnSO_4_ aqueous solution using Zn as anode [46]. Copyright 2021, American Chemical Society

Third, optimizing the composition of electrolyte salts also was very effective in enhancing the electrochemical performance of batteries. Jiang et al. used an inert-cation-assisted water-in-salt (IC-WiS) electrolyte to improve the electrochemical performance of Na_1.88_Mn[Fe(CN)_6_]_0.97_·1.35H_2_O (NaMnHCF) cathode [123]. The IC-WiS electrolyte consisted of 9 M sodium triflate (NaOTF) and 22 M tetraethylammonium triflate (TEAOTF), which expanded the electrochemical window to 3.3 V. The IC-WiS electrolyte could suppress the dissolution of transition metal from NaMnHCF electrode and avoid the mixed-cation co-intercalation because of the larger radius of the TEA^+^ cation, which could improve cycling performance of battery. The NaMnHCF electrode showed a first discharge capacity of 140 mAh g^− 1^ at 1C in both 9 M NaOTF electrolyte and 9 M NaOTF + 22 M TEAOTF electrolyte, as shown in Fig. 15a. However, after 50 cycles, the discharge capacities decreased to 84 and 137 mAh g^− 1^ for 9 M NaOTF electrolyte and 9 M NaOTF + 22 M TEAOTF electrolyte, respectively. In the 9 M NaOTF + 22 M TEAOTF electrolyte, the NaMnHCF electrode exhibited superior cycling stability with negligible capacity loss. A NaMnHCF|IC-WiS|NaTiOPO_4_ full battery displayed a discharge capacity of 41 mAh g^− 1^ (based on cathode and anode) at 0.25C (Fig. 15b), corresponding to 71 Wh kg^− 1^ energy density. The full battery also delivered excellent cycling stability with capacity retention of 90% after 200 cycles at 0.25C and limited self-discharge behavior (Fig. 15c). In addition, the anion in water-in-salt electrolytes affects tremendously solution structure and electrochemical stability in aqueous high-voltage batteries. Reber et al. compared systematically water-in-salt electrolytes based on 11 sodium salts [124]. Multisolvent systems and ternary electrolytes could improve the cycling stability. A Na_2_Mn[Fe(CN)_6_]|NaTi_2_(PO_4_)_3_ full cell based on a highly concentrated electrolyte with a mixed water/ionic liquid solvent system presented excellent cycling performance. The full cell showed high coulombic efficiency of 99.8% and capacity retention of 79% after 300 cycles at 1C in 80 M NaTFSI/EMImTFSI (TFSI: bis(trifluoromethanesulfonyl)imide; EMIm: 1-ethyl-3-methylimidazolium) electrolyte, as shown in Fig. 15d.

All in all, optimizing aqueous electrolyte by choosing suitable electrolyte additive, adjusting electrolyte salt concentration and adopting suitable sodium salts (one or a combination of multiple sodium salts) could effectively improve the reversible capacity and cycling performance of Mn-based Prussian blue analogues.

(2) **Optimization of Vacancies in Prussian Blue Analogues**

The presence of vacancies in Prussian blue analogues affects their stability. Reguera et al. investigated the effect of vacancies on electrochemical performance of Na_x_Co_1–y_Mn_y_[Fe(CN)_6_] [125]. Na_1.88_Co_0.55_Mn_0.45_[Fe(CN)_6_]_0.97_ (labeled as Co_0.55_Mn_0.45_HF) material without vacancies was synthesized by precipitation method using citrate as chelating agent. For comparison, a similar compound Na_1.65_Mn_0.50_Co_0.50_[Fe(CN)_6_]_0.87_ (labeled as *n*-CoMnHF) with [Fe(CN)_6_]^4−^ vacancies was also prepared. As shown in Fig. 15e, the *n*-CoMnHF electrode in 1 M NaNO_3_ solution exhibited a lower discharge capacity of 87 mAh g^− 1^ than the Co_0.55_Mn_0.45_HF electrode (112.82 mAh g^− 1^), due to the presence of [Fe(CN)_6_]^4−^ vacancies. The Co_0.55_Mn_0.45_HF electrode showed high electrochemical stability with capacity retention of 80% after 100 cycles, while the *n*-CoMnHF electrode only displayed 60% capacity retention (Fig. 15f). The improvement in electrochemical stability of Co_0.55_Mn_0.45_HF without vacancies could be related to electronic interaction between external metals. In a vacancy-free framework, the charge transfer between Fe and Mn was disrupted due to the interaction between Mn and Co, which improved the electrochemical properties. Therefore, reducing the content of vacancies in Mn-based Prussian blue analogues could greatly enhance their electrochemical performance.

(3) **Carbon Modification**

Graphene oxide (GO) and RGO were adopted to improve the electrochemical performance of Mn-based Prussian blue analogues. Zhang et al. investigated the electrochemical performance of GO or RGO-modified Na_2_MnFe(CN)_6_ (PBM) cathode materials in 1 M Na_2_SO_4_ + 1 M ZnSO_4_ aqueous solution using Zn anode [46]. The initial discharge capacities were 71.0, 109.5 and 115.4 mAh g^− 1^ at 85 mA g^− 1^ for PBM, GO/PBM and RGO/PBM electrodes (Fig. 15g), respectively. The capacity retention was 57.5% after 200 cycles for PBM electrode. In contrast, after 200 cycles, the GO/PBM and RGO/PBM electrodes delivered high capacity retention with discharge capacities of 100.5 and 102.2 mAh g^− 1^, respectively. In addition, the PBM electrode showed poor rate capability with a discharge capacity of 7 mAh g^− 1^ at 500 mA g^− 1^. On the contrary, the GO/PBM and RGO/PBM electrodes displayed excellent rate capability (Fig. 15h), and the discharge capacities were 66 and 79 mAh g^− 1^ at 500 mA g^− 1^ for GO/PBM and RGO/PBM electrodes, respectively. An energy density of 165 Wh kg^− 1^ was achieved for RGO/PBM|Zn battery. The improvement of electrochemical performance could be attributed to the coating of GO or RGO film on PBM, which could increase electronic conductivity, prevent structure collapse and make the PBM material more stable.

In short, Mn-based Prussian blue analogues, as suitable cathode materials for aqueous SIBs, could show good electrochemical performance and high energy density by optimizing electrolyte, using carbon modification, and reducing vacancies. The electrochemical performance of the Mn-based Prussian blue analogues introduced previously is summarized in Table 2.

**Table 2:** Electrochemical properties of the Mn-based Prussian Blue analogues and Mn-based polyanion compounds for aqueous SIBs

Working electrode	Counter electrode	Electrolyte	Voltage range (V)	Capacity (mAh g^− 1^) / Rate (mA g^− 1^)	Capacity retention (%) (cycles)	Refs.
Na_2_MnFe(CN)_6_	NaTi_2_(PO_4_)_3_	1 M Na_2_SO_4_	–	85 (-)	78.6 (30)	[42]
Na_2_MnFe(CN)_6_	AC	8 M CH_3_COONa- 32 M CH_3_COOK	0–1.0	75 (100)	–	[116]
Na_2_MnFe(CN)_6_	AC	17 M NaClO_4_	0–1.5	119 (500)	92.5 (100)	[119]
Na_2_MnFe(CN)_6_	Pt	1 M Na_2_SO_4_-1 M ZnSO_4_ + SDS	0.2–1.2	140 (80)	–	[22]
Na_2_MnFe(CN)_6_	Zn	1 M Na_2_SO_4_-1 M ZnSO_4_ + SDS	1.0–2.0	137 (80)	75 (2000)	[22]
Na_2_MnFe(CN)_6_	NaTi_2_(PO_4_)_3_	17 M NaClO_4_	0.5–2.0	117 (-)	81 (50)	[122]
Na_1.88_Mn[Fe(CN)_6_]_0.97_·1.35H_2_O	AC	9 M NaOTF-22 M TEAOTF	0–1.3	140 (140)	No decay (50)	[123]
Na_1.88_Co_0.55_Mn_0.45_[Fe(CN)_6_]_0.97_	C	1 M NaNO_3_	0–1.25	113 (60)	80 (100)	[125]
Na_1.65_Mn_0.50_Co_0.50_[Fe(CN)_6_]_0.87_	C	1 M NaNO_3_	0–1.2	87 (60)	60 (100)	[125]
Na_1.33_Mn[Fe(CN)_6_]_0.79_·1.88H_2_O	AC	10 M NaClO_4_	0.5–1.5	125 (120)	–	[121]
Na_2_MnFe(CN)_6_	Zn	1 M Na_2_SO_4_-1 M ZnSO_4_	0.9–2.0	71 (85)	57.5 (200)	[46]
GO/Na_2_MnFe(CN)_6_	Zn	1 M Na_2_SO_4_-1 M ZnSO_4_	0.9–2.0	109.5 (85)	91.8 (200)	[46]
RGO/Na_2_MnFe(CN)_6_	Zn	1 M Na_2_SO_4_-1 M ZnSO_4_	0.9–2.0	115.4 (85)	88.6 (200)	[46]
Na_3_MnTi(PO_4_)_3_	Na_3_MnTi(PO_4_)_3_	1 M Na_2_SO_4_	0.4–1.8	57.9 (29.3)	98 (100)	[43]
Na_4_MnV(PO_4_)_3_-RGO	C	10 M NaClO_4_	0–0.82	92 (110)	–	[133]
Na_4_MnV(PO_4_)_3_-RGO	NaTi_2_(PO_4_)_3_-MWCNT	10 M NaClO_4_	0.8–1.65	97 (1100)	51.5 (100)	[133]
Na_3_MnPO_4_CO_3_	Zn	17 M NaClO_4_	-1.2–1.3	134 (-)	55 (30)	[134]
Na_3_MnPO_4_CO_3_	NaTi_2_(PO_4_)_3_	5 M NaNO_3_	0–1.5	77.09 (-)	78 (100)	[136]
Mn_2_(PO_4_)F-CeO_2_	Zn	4 M NaClO_4_	0–2.0	104 (12)	99 (300)	[137]
Mn_2_(PO_4_)F–C-CeO_2_	Zn	4 M NaClO_4_	0–2.0	195 (12)	78 (300)	[137]

### Polyanion Compounds

Polyanion compounds are among the most promising cathode materials for SIBs, because of their safety, stability and suitable operating voltages [126, 127]. Many polyanion compounds have been investigated for aqueous SIBs [128, 129]. However, there are only a few Mn-based polyanion compounds reported as cathodes for aqueous SIBs, including NaMn_1/3_Co_1/3_Ni_1/3_PO_4_, Na_3_MnTi(PO_4_)_3_, Na_4_MnV(PO_4_)_3_, Na_3_MnPO_4_CO_3_ and Mn_2_(PO_4_)F.

1. **Mn-based Phosphates**

**Fig. 16:**
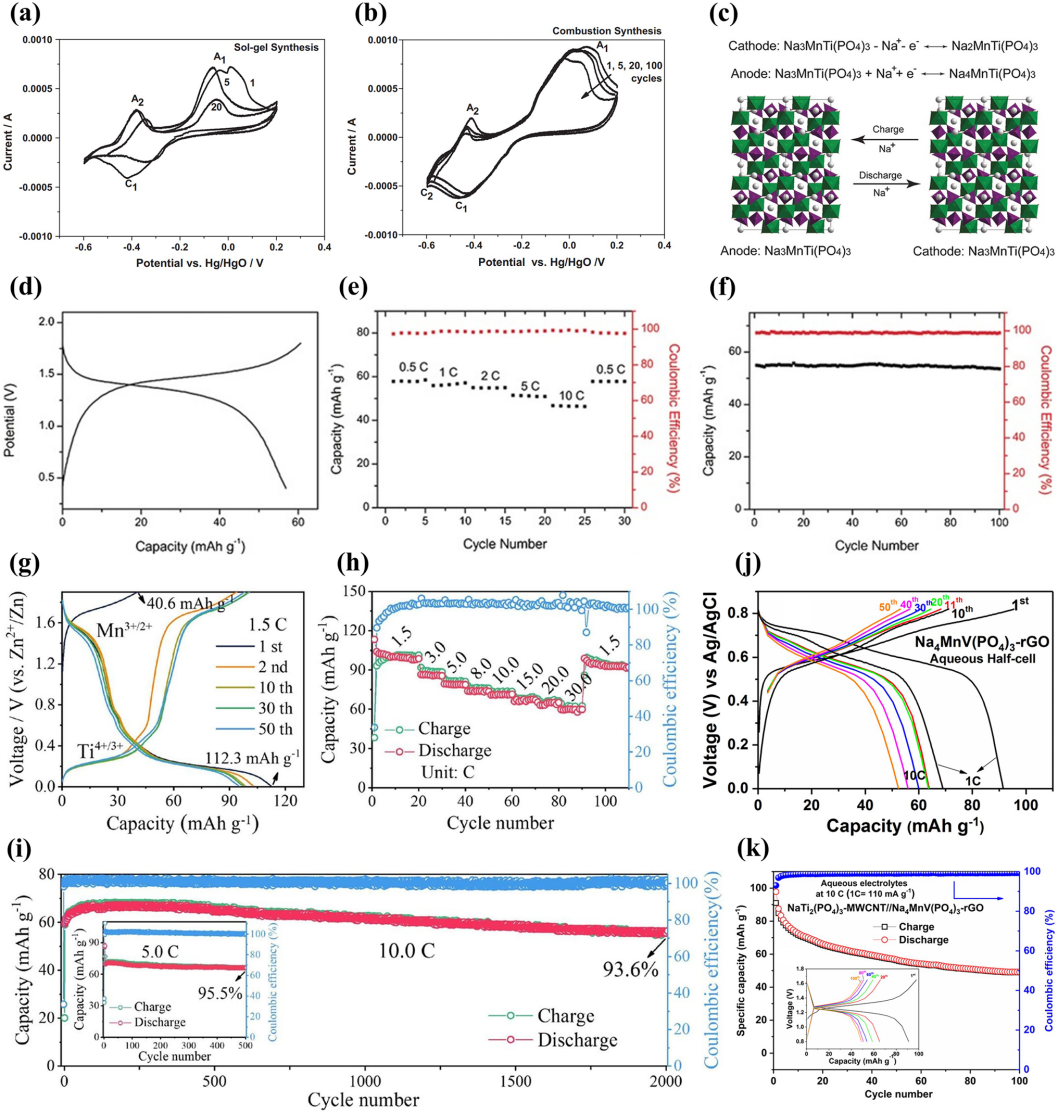
CV curves of NaMn_1/3_Co_1/3_Ni_1/3_PO_4_ electrodes synthesized by** a** sol–gel and **b** combustion method [130]. Copyright 2012, Elsevier. **c** Schematic illustration of aqueous symmetric sodium-ion battery with Na_3_MnTi(PO_4_)_3_ as anode and cathode. **d** Charge/discharge curve at 0.5C, **e** rate performance and** f** cycling performance at 1C of the symmetric battery with Na_3_MnTi(PO_4_)_3_ as anode and cathode in 1 M Na_2_SO_4_ aqueous solution [43]. Copyright 2016, Wiley‐VCH. **g** Charge/discharge profiles at 1.5C, **h** rate capability and **i c**ycling performance at 10C of Na_3_MnTi(PO_4_)_3_|Zn battery with 0.5 M CH_3_COONa and Zn(CH_3_COO)_2_ mixed aqueous electrolyte at 0.01–1.9 V [131]. Copyright 2021, Elsevier.** j** Charge–discharge profiles for Na_4_MnV(PO_4_)_3_-RGO electrode in 10 M NaClO_4_ (with 2 vol% VC). **k** Cycling performance at 10C for Na_4_MnV(PO_4_)_3_-RGO|NaTi_2_(PO_4_)_3_-MWCNT full cell in 10 M NaClO_4_ (with 2 vol% VC) electrolyte [133]. Copyright 2019, Elsevier

Minakshi et al. synthesized NaMn_1/3_Co_1/3_Ni_1/3_PO_4_ cathode materials by sol–gel and combustion routes, and investigated their electrochemical properties in 7 M NaOH solution using Zn as counter electrode [130]. For NaMn_1/3_Co_1/3_Ni_1/3_PO_4_ electrode electrodes, the electrochemical redox process was fully reversible. The NaMn_1/3_Co_1/3_Ni_1/3_PO_4_ electrode prepared by sol–gel route showed only 55% cyclic efficiency after 20 cycles (Fig. 16a). In contrast, the NaMn_1/3_Co_1/3_Ni_1/3_PO_4_ electrode prepared by combustion route delivered excellent cyclic efficiency and cycling stability, with 87% efficiency after 100 cycles (Fig. 16b). As a result, Na ions diffusion into the NaMn_1/3_Co_1/3_Ni_1/3_PO_4_ might be affected by the synthesis technique and associated physical properties.

Besides, NASICON-structured Na_3_MnTi(PO_4_)_3_ materials have also been explored. Gao et al. reported a symmetric battery using NASICON-structured Na_3_MnTi(PO_4_)_3_ as cathode and anode (Fig. 16c) [43]. A three-dimensional framework was formed in Na_3_MnTi(PO_4_)_3_, where MnO_6_ or TiO_6_ octahedra was sharing its corners with PO_4_ tetrahedra. The insertion/extraction of Na ions in Na_3_MnTi(PO_4_)_3_ could occur by the redox couples of Mn^3+^/Mn^2+^ and Ti^4+^/Ti^3+^ in 1 M Na_2_SO_4_ aqueous solution. The symmetric cell with 1 M Na_2_SO_4_ electrolyte delivered a reversible discharge capacity of 57.9 mAh g^− 1^ at 0.5C (1C = 58.7 mA g^− 1^) (Fig. 16d), and had an energy density of about 40 Wh kg^− 1^ (based on anode and cathode). The symmetric cell also demonstrated excellent rate capability with a discharge capacity of 46.7 mAh g^− 1^ at 10C (Fig. 16e) and excellent cycling performance with coulombic efficiency exceeding 99% and capacity retention of 98% after 100 cycles at 1C (Fig. 16f). After that, Zhou et al. investigated a hybrid sodium/zinc battery based on Na_3_MnTi(PO_4_)_3_ cathode and Zn anode with 0.5 M CH_3_COONa and Zn(CH_3_COO)_2_ mixed aqueous electrolyte [131]. The first charge profile delivered a charge capacity of 40.6 mAh g^− 1^ at 1.5C (175.5 mA g^− 1^) with only one charge plateau (Fig. 16g), and the subsequent discharge–charge profiles showed two reversible voltage plateaus corresponding to insertion/extraction reaction of Na ions, with an initial discharge capacity of 112.3 mAh g^− 1^. The Na_3_MnTi(PO_4_)_3_|Zn battery also demonstrated outstanding rate capability and the discharge capacities were 86.5, 70.9, 65.3 and 59.8 mAh g^− 1^ at 3C, 10C, 20C and 30C (Fig. 16h), respectively. More importantly, the Na_3_MnTi(PO_4_)_3_|Zn battery displayed superior cycling performance with capacity retention of 93.6% after 2000 cycles (Fig. 16i), indicating good structural stability of Na_3_MnTi(PO_4_)_3_ electrode. Very recently, wu et al. reported that the reversible discharge capacity of Na_3_MnTi(PO_4_)_3_ could be improved by increasing Mn content [132]. The discharge capacity of Na_3.4_Mn_1.2_Ti_0.8_(PO_4_)_3_ was 68.2 mAh g^− 1^ at 100 mA g^− 1^ in 1 M Na_2_SO_4_, which was higher than that (52.6 mAh g^− 1^) of Na_3_MnTi(PO_4_)_3_. However, a capacity reduction occurred when Mn content increased to form Na_3.8_Mn_1.4_Ti_0.6_(PO_4_)_3_, which showed a discharge capacity of 60.8 mAh g^− 1^. It might be caused by the impurity phase in the sample and some side reactions.

Furthermore, Na_4_MnV(PO_4_)_3_-RGO composites were developed as cathode by Kumar et al. [133]. The Na_4_MnV(PO_4_)_3_-RGO electrode in 10 M NaClO_4_ (with 2 vol% vinylene carbonate) using carbon paper as counter electrode delivered a discharge capacity of 92 mAh g^− 1^ at 1C (110 mA g^− 1^) with 82% capacity retained after 10 cycles, and a stable discharge capacity of 60 mAh g^− 1^ at 10C for 40 cycles (Fig. 16j). A full cell Na_4_MnV(PO_4_)_3_-RGO|NaTi_2_(PO_4_)_3_-MWCNT showed an initial discharge capacity of 97 mAh g^− 1^ at 10C and 51.5% capacity retention after 100 cycles, as shown in Fig. 16k. The enhanced electrochemical performance could be ascribed to the improved electronic conductivity in the RGO network, which was homogenously integrated with Na_4_MnV(PO_4_)_3_ particles.

In brief, the Mn-based phosphates, especially NASICON-structured Na_3_MnTi(PO_4_)_3_ materials, show better electrochemical performance. More efforts should be made to explore new Mn-based phosphates for aqueous SIBs. Some improvement methods should be adopted to further enhance their electrochemical performance.

(2) **Mn-based Mixed-Polyanions**

**Fig. 17:**
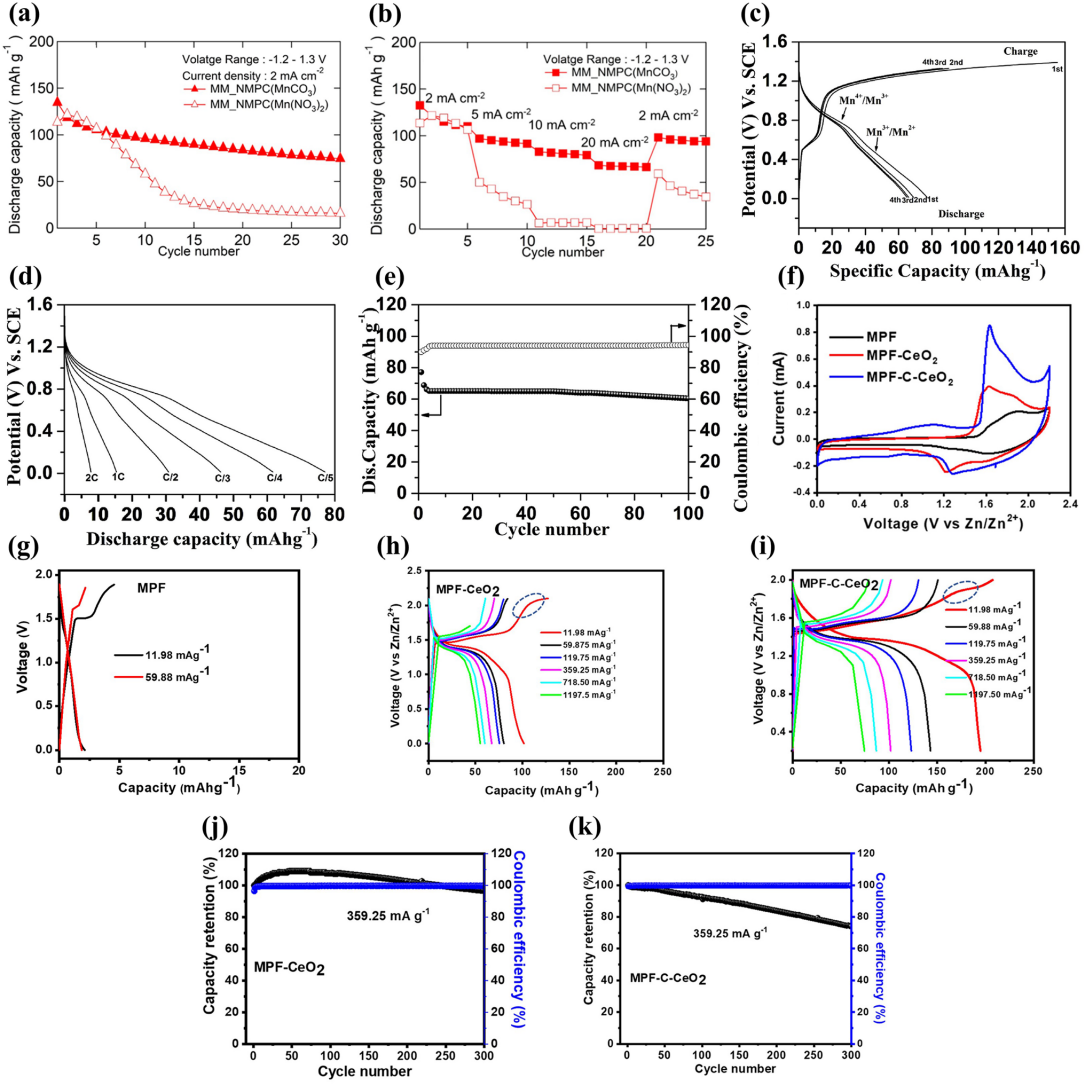
**a** Cycling performance and **b** Rate capability of Na_3_MnPO_4_CO_3_ electrodes with various starting materials (MM_NMPC (MnCO_3_) and MM_NMPC (Mn(NO_3_)_2_)) [134]. Copyright 2019, The authors. **c** Charge–discharge curves at C/5, **d** discharge curves at various C rates and **e** cycling performance at C/5 of Na_3_MnPO_4_CO_3_|NaTi_2_(PO_4_)_3_ full cell in 5 M NaNO_3_ aqueous electrolyte [136]. Copyright 2020, Elsevier. **f** CV curves of MFP, MFP-CeO_2_ and MFP-C-CeO_2_ electrodes in 4 M NaClO_4_ aqueous electrolyte using Zn as anode at 2.5 mV s^− 1^ (after 5 charge–discharge cycles at 12 mA g^− 1^). Charge–discharge curves at different current density of **g** MPF electrode, **h** MPF-CeO_2_ electrode and **i** MPF-C-CeO_2_ electrode. Cycling performance at 359.25 mA g^− 1^ of **j** MPF-CeO_2_ electrode and **k** MPF-C-CeO_2_ electrode [137]. Copyright 2021, American Chemical Society

Mixed-polyanions Na_3_MnPO_4_CO_3_ materials were developed as cathode materials for aqueous SIBs [134–136]. Xie et al. synthesized Na_3_MnPO_4_CO_3_ by mechanical ball milling method and investigated its electrochemical performance in 17 M NaClO_4_ aqueous solution using Zn as anode [134]. The Na_3_MnPO_4_CO_3_ synthesized from Na_3_PO_4_ and MnCO_3_ was denoted as MM_NMPC (MnCO_3_), and the average particle size (d_50_) was 5.6 μm. The Na_3_MnPO_4_CO_3_ synthesized from Mn(NO_3_)_2_·4H_2_O, Na_2_HPO_4_·2H_2_O and Na_2_CO_3_·H_2_O was denoted as MM_NMPC (Mn(NO_3_)_2_), and the d_50_ was 12.7 μm. The MM_NMPC (MnCO_3_) electrode delivered a discharge capacity of 134 mAh g^− 1^ at 2 mA cm^−2^, and had a retention capacity of near 74 mAh g^− 1^ after 30 cycles (Fig. 17a). However, the MM_NMPC (Mn(NO_3_)_2_) electrode displayed only a first discharge capacity of 113 mAh g^− 1^ and had a retention capacity of near 16 mAh g^− 1^ after 30 cycles, which could be caused by its larger overvoltage resulting from larger particle size. Compared with MM_NMPC (Mn(NO_3_)_2_) electrode, the MM_NMPC (MnCO_3_) electrode also exhibited better rate capability with a specific capacity of 68 mAh g^− 1^ at 20 mA cm^−2^ (Fig. 17b). Furthermore, Shiprath et al. synthesized Na_3_MnPO_4_CO_3_ nanoparticles with average particle size of 20–30 nm by low temperature ionothermal method [136]. A Na_3_MnPO_4_CO_3_|NaTi_2_(PO_4_)_3_ full cell constructed using 5 M NaNO_3_ aqueous electrolyte delivered a low discharge capacity of 77.09 mAh g^− 1^ at C/5 (1C = 191 mA g^− 1^) in the first cycle (Fig. 17c), and the lower capacity could be caused by poor electronic conductivity of Na_3_MnPO_4_CO_3_. The full cell had poor rate capability with a discharge capacity of about 31 mAh g^− 1^ at C/2 (Fig. 17d) and exhibited good cycling performance with about 78% capacity retention after 100 cycles (Fig. 17e). In short, the mixed-polyanions Na_3_MnPO_4_CO_3_ materials exhibit high reversible capacity but have poor rate capability and cycling performance, and some optimization strategies should be developed to improve their electrochemical performance.

(3) **Mn-based Fluorophosphates**

Fluorophosphates, Mn_2_(PO_4_)F (MFP), as cathode material was reported by Nzimande et al. [137]. MFP, ceria-coated MFP (MFP-CeO_2_) and ceria- and carbon-coated MFP (MFP-C-CeO_2_) were synthesized by microwave-assisted hydrothermal process. As shown in Fig. 17f, the redox behavior of MPF in 4 M NaClO_4_ aqueous electrolyte was improved by ceria coating and ceria-carbon coating. The MFP electrode displayed extremely poor charge–discharge response with a reversible discharge capacity of 2 mAh g^− 1^ at 12 mA g^− 1^ (Fig. 17g). With ceria coating, the MFP-CeO_2_ electrode exhibited a discharge capacity of 104 mAh g^− 1^ at 12 mA g^− 1^ and excellent rate capability with a discharge capacity of 58 mAh g^− 1^ at 1198 mA g^− 1^ (Fig. 17h). With ceria-carbon coating, the MFP-C-CeO_2_ electrode showed a discharge capacity of 195 mAh g^− 1^ at 12 mA g^− 1^ and good rate capability with a discharge capacity of 60 mAh g^− 1^ at 1198 mA g^− 1^ (Fig. 17i). However, the MFP-CeO_2_ electrode demonstrated excellent cycling stability with capacity retention of 99% after 300 cycles (Fig. 17j) compared to the MFP-C-CeO_2_ electrode (78% capacity retention) (Fig. 17k). The ceria coating could stable the electrode structure, improve ionic conductivity of electrode, protect electrode from the etching effect of electrolyte, and induce catalytic activity, which enhanced the redox behavior of electrode. Therefore, ceria coating and carbon coating can be used to further enhance the electrochemical performance of electrode by optimizing the carbon/ceria content.

Based on the above discussion, it can be found that different Mn-based polyanion compounds show various electrochemical performance, which were affected by compound composition, particle size, carbon modification and electrolyte solution. The electrochemical performance of the Mn-based polyanion compounds mentioned above is summarized in Table 2.

## Mn-based Anode Materials

There are only a few Mn-based materials investigated as anode materials for aqueous SIBs, including oxides, Prussian blue analogues and polyanion compounds.

### Oxides

As cathode materials, *δ*-MnO_2_, Na_0.27_MnO_2_ and Mn_5_O_8_ have been introduced. At the same time, these materials were also used as anode materials to assemble symmetric full cells. A (Ni)MnO_2_|Na_2_SO_4_(1 M)|(Ni)MnO_2_ full cell delivered a discharge capacity of 63 mAh g^− 1^ at 200 mA g^− 1^ and superior cycle stability without capacity loss over 2000 cycles [55]. Similarly, a Na_0.27_MnO_2_|Na_2_SO_4_(1 M)|Na_0.27_MnO_2_ full cell demonstrated a discharge capacity of 83 mAh g^− 1^ at 1000 mA g^− 1^ and excellent cycling performance without obvious capacity loss over 5000 cycles [64]. Moreover, a higher discharge capacity of 103 mAh g^− 1^ at 5000 mA g^− 1^ was obtained for Mn_5_O_8_|Na_2_SO_4_(1 M)|Mn_5_O_8_ full cell, which also showed excellent cycling performance without capacity fade upon 5000 cycles [113]. Based on these oxides, the symmetric full cells exhibited high reversible and excellent cycling performance.

**Fig. 18:**
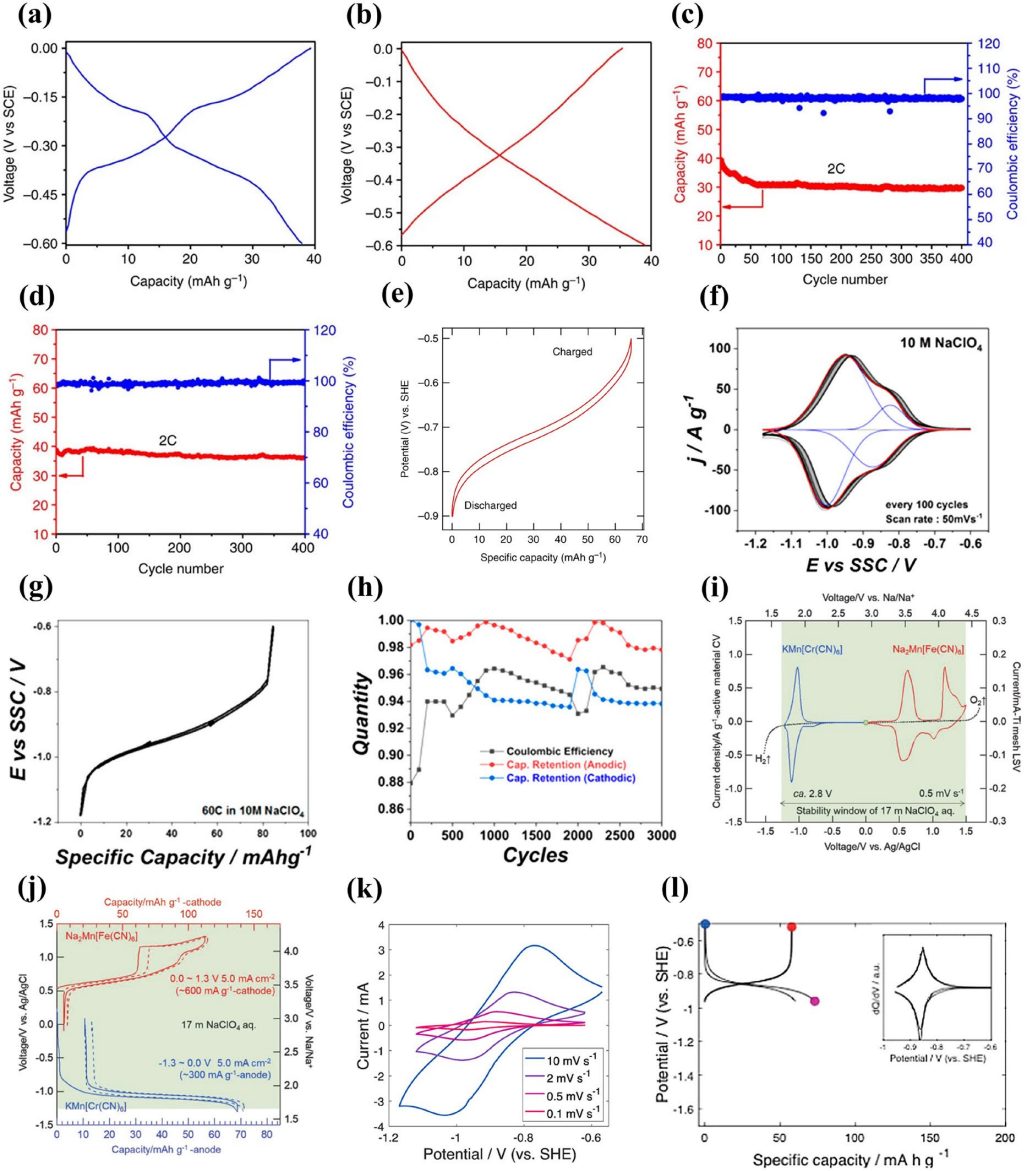
Charge/discharge curves in the first cycle at 2C for **a** Na_0.44_MnO_2_ electrode and **b** Na_0.44_[Mn_0.44_Ti_0.56_]O_2_ electrode in Na_2_SO_4_ aqueous electrolytes (pH = 13.5) using platinum electrode as counter electrode. Cycling performance at 2C for **c** Na_0.44_MnO_2_ electrode and **d** Na_0.44_[Mn_0.44_Ti_0.56_]O_2_ electrode in Na_2_SO_4_ aqueous electrolytes [138]. Copyright 2015, Springer Nature. **e** Charge–discharge profile of Na_1.24_Mn[Mn(CN)_6_]_0.81_·2.1H_2_O electrode vs. SHE at 60 mA g^− 1^ in cosolvent electrolyte (1 M NaClO_4_, 90% acetonitrile, 10% water) [141]. Copyright 2018, The Author(s).** f** CV curves at 50 mV s^− 1^ of Na_x_Mn[Mn(CN)_6_] thin film in 10 M NaClO_4_ solution using Pt as counter electrode for over 3000 cycles (every 100th cycle shown). **g** Charge–discharge profile of Na_x_Mn[Mn(CN)_6_] thin film in 10 M NaClO_4_ solution at 60C. **h** Cathodic and anodic capacity retention of Na_x_Mn[Mn(CN)_6_] thin film [142]. Copyright 2018, American Chemical Society.** i** CV curves of Na_2_Mn[Fe(CN)_6_] and KMHCC (KMn[Cr(CN)_6_]) electrodes in 17 M NaClO_4_ aqueous electrolyte. **j** Charge/discharge curves of Na_2_Mn[Fe(CN)_6_] and KMHCC (KMn[Cr(CN)_6_]) electrodes [143]. Copyright 2018, Wiley‐VCH.** k** CV curves of NMHCC electrode in 37 M NaFSI aqueous solution with manganese Hexacyanoferrate as counter electrode. **m** Charge–discharge profiles of NMHCC electrode at 15 mA g^− 1^ [144]. Copyright 2019, American Chemical Society

Apart from these oxides, Wang et al. investigated Ti-substituted Na_0.44_MnO_2_ (Na_0.44_[Mn_1-*x*_Ti_*x*_]O_2_) as anode material [138]. The initial discharge capacities were about 37 and 39 mAh g^− 1^ at 2C (100 mA g^− 1^) for Na_0.44_MnO_2_ and Na_0.44_[Mn_0.44_Ti_0.56_]O_2_ electrodes in Na_2_SO_4_ aqueous electrolytes (Fig. 18a-b), respectively. The Na_0.44_MnO_2_ electrode displayed a stable capacity of 32 mAh g^− 1^ after 50 cycles and capacity retention of 86.5% after 400 cycles (Fig. 18c). Compared to the Na_0.44_MnO_2_ electrode, the Na_0.44_[Mn_0.44_Ti_0.56_]O_2_ electrode demonstrated excellent cycling performance with capacity retention of 95% after 400 cycles (Fig. 18d). However, compared to *δ*-MnO_2_, Na_0.27_MnO_2_ and Mn_5_O_8_, the Ti-substituted Na_0.44_MnO_2_ had lower reversible capacity and poor cycling stability. More recently, Na_2_[Mn_3_Vac_0.1_Ti_0.4_]O_7_ (Vac represents vacancy) was studied as anode material for aqueous SIBs [139]. A full cell Na_0.44_MnO_2_|Na_2_[Mn_3_Vac_0.1_Ti_0.4_]O_7_ using 9 M NAOTF + 22 M TEAOTF aqueous electrolyte delivered a reversible capacity of 57.4 mAh g^− 1^ at 2C (based on the mass of anode active material). The reversible capacity of Na_2_[Mn_3_Vac_0.1_Ti_0.4_]O_7_ was still lower. Therefore, among all the Mn-based oxides as anode materials, Mn_5_O_8_ material exhibits the best electrochemical performance.

### Prussian Blue Analogues

Prussian blue analogues have exhibited excellent electrochemical performance as cathode materials for aqueous SIBs. Due to their large channels and interstices, Prussian blue analogues were also investigated as anode materials for aqueous SIBs.

1. **Manganese Hexacyanomanganate**

Pasta et al. reported a manganese hexacyanomanganate open-framework anode, K_0.11_Mn[Mn(CN)_6_]_0.83_·□_0.17_·3.64H_2_O (□ = Mn(CN)_6_ vacancy) [140]. A full cell assembled with copper hexacyanoferrate cathode, manganese hexacyanomanganate anode and 10 M NaClO_4_ saturated with Mn(ClO_4_)_2_ as electrolyte had an average discharge voltage of 0.95 V and delivered a specific capacity of about 26 mAh g^− 1^ at 10C. In particular, the full cell showed high rate capability with 73.3% capacity retention at 50C and excellent cycling performance with no capacity loss after 1000 cycles. Moreover, Firouzi et al. investigated Na_1.24_Mn[Mn(CN)_6_]_0.81_·2.1H_2_O as anode for aqueous SIBs [141]. The Na_1.24_Mn[Mn(CN)_6_]_0.81_·2.1H_2_O electrode delivered a specific capacity of 67 mAh g^− 1^ at 60 mA g^− 1^ in cosolvent electrolyte (1 M NaClO_4_, 90% acetonitrile, 10% water) (Fig. 18e). The Na_1.24_Mn[Mn(CN)_6_]_0.81_·2.1H_2_O electrode also displayed excellent cycling stability with a specific capacity of 66 mAh g^− 1^ at 60 mA g^− 1^ after 700 cycles corresponding to 98.5% capacity retention. In addition, Yun et al. developed Na_x_Mn[Mn(CN)_6_] thin films as anode materials [142]. The Na_*x*_Mn[Mn(CN)_6_] thin film showed very low half-charge potential of about − 0.73 V vs SHE (− 0.93 V vs SSC), which could inhibit hydrogen evolution reaction. Figure 18f displays the CV curves at 50 mV s^− 1^ of Na_x_Mn[Mn(CN)_6_] thin film in 10 M NaClO_4_ solution using Pt as counter electrode for over 3000 cycles (every 100th cycle shown). There was no hydrogen evolution reaction observed. After 100 cycles, the anodic and cathodic half-charged potentials were as low as − 0.930 and − 0.958 V, respectively, showing superior cycling stability. A specific discharge capacity of about 85 mAh g^− 1^ was obtained at 60C (5 A g^− 1^) (Fig. 18g). The Na_x_Mn[Mn(CN)_6_] thin film also exhibited superior cycling stability with capacity retention of 97% over 3000 cycles (Fig. 18h).

(2) ** Manganese Hexacyanochromate**

Nakamoto et al. investigated manganese hexacyanochromate, K_0.01_Mn[Cr(CN)_6_]_0.72_·2.01H_2_O (KMHCC), as anode material [143]. Figure 18i shows the CV curve at 0.5 mV s^− 1^ of KMHCC anode in 17 M NaClO_4_ aqueous electrolyte, and a pair of symmetrical redox peaks and few irreversible reduction peaks could be observed. The KMHCC anode exhibited one voltage plateau at − 1.1 V and a discharge capacity of about 58 mAh g^− 1^ at 5.0 mA cm^−2^ (Fig. 18j). A full cell with KMHCC anode, Na_2_MnFe(CN)_6_ cathode and 17 M NaClO_4_ aqueous electrolyte demonstrated a discharge capacity of about 37 mAh g^− 1^ at 5C (200 mA g^− 1^). At high rate of 60C, a capacity of 16 mAh g^− 1^ was maintained, indicating high rate capability. However, the full cell showed poor cycling performance at low rate of 5C and good cyclability at high rate of 30C. In addition, manganese hexacyanochromate, Na_0.04_Mn[Cr(CN)_6_]_0.70_·2.80H_2_O (NMHCC), with the lowest redox potential was studied by Wheeler et al. [144]. Figure 18k shows the CV curves of NMHCC electrode in 37 M sodium bis(fluorosulfonyl)imide (NaFSI) aqueous solution with manganese hexacyanoferrate as counter electrode. The increase of peak separation and peak current with an increase in scan rate indicated a one-electron one-step quasi-reversible reaction. There was a voltage plateau centered at − 0.86 V in the charge–discharge profiles, and a reversible capacity of 62 mAh g^− 1^ at 15 mA g^− 1^was obtained for NMHCC electrode (Fig. 18m).

In a word, manganese hexacyanomanganate presents high reversible capacity and superior cycling stability compared with manganese hexacyanochromate. Among all the Mn-based Prussian blue analogues anodes, Na_x_Mn[Mn(CN)_6_] exhibits the best electrochemical performance. The electrochemical performance is affected by aqueous electrolyte and composition and vacancies of Prussian blue analogues, and some improvement approaches should be adopted to enhance the electrochemical performance.

### Polyanion Compounds

NASICON-type NaTi_2_(PO_4_)_3_ with an open framework is a typical anode material for aqueous SIBs [20, 145]. Some Mn-based polyanion compounds were also investigated as anode materials. NASICON-type Na_3_MnTi(PO_4_)_3_ was reported by Gao et al. as anode and cathode materials, and a Na_3_MnTi(PO_4_)_3_|Na_3_MnTi(PO_4_)_3_ symmetric cell with 1 M Na_2_SO_4_ electrolyte showed a reversible discharge capacity of 57.9 mAh g^− 1^ at 29.35 mA g^− 1^ [43]. The symmetric cell also demonstrated excellent rate capability and cycling performance with capacity retention of 98% after 100 cycles. Furthermore, Na_2_Ti_3/2_Mn_1/2_(PO_4_)_3_ nanodots planted in carbon matrix was also reported as low-cost anode by Lei et al. [146]. The Na_2_Ti_3/2_Mn_1/2_(PO_4_)_3_ material displayed an initial discharge capacity of 88.6 mAh g^− 1^ at 0.5C in 6 M NaClO_4_ aqueous electrolyte using nickel hexacyanoferrate as counter electrode. Owing to ultrafast Na-intercalation chemistry, the Na_2_Ti_3/2_Mn_1/2_(PO_4_)_3_ material had excellent high rate performance with stable capacity of 65.1 mAh g^− 1^ at 10C and stable cycling performance (90% capacity retention after 1000 cycles at 10C). Therefore, as anode materials, Mn-based polyanion compounds demonstrated excellent electrochemical performance, although there were only a few materials reported.

From the above discussion, it can be found that different types of Mn-based anode materials showed diverse electrochemical performance, and oxides exhibited better electrochemical performance. A comprehensive summary of the electrochemical performance of the Mn-based anode materials introduced previously is presented in Table 3.

**Table 3:** Electrochemical properties of the Mn-based anode materials for aqueous SIBs

Working electrode	Counter electrode	Electrolyte	Voltage range (V)	Capacity (mAh g^− 1^) / Rate (mA g^− 1^)	Capacity retention (%) (cycles)	Refs.
Ni-doped *δ*-MnO_2_	Ni-doped *δ*-MnO_2_	1 M Na_2_SO_4_	0–1.6	63 (200)	No decay (2000)	[55]
Na_0.27_MnO_2_	Na_0.27_MnO_2_	0.5 M Na_2_SO_4_	0–2.5	83 (1000)	No decay (5000)	[64]
Mn_5_O_8_	Mn_5_O_8_	1 M Na_2_SO_4_	0–3.0	103 (5000)	No decay (5000)	[113]
Na_0.44_MnO_2_	Pt	Na_2_SO_4_ (pH = 13.5)	-0.6–0	37 (100)	86.5 (400)	[138]
Na_0.44_[Mn_1-*x*_Ti_*x*_]O_2_	Pt	Na_2_SO_4_ (pH = 13.5)	− 0.6–0	39 (100)	95 (400)	[138]
K_0.11_Mn[Mn(CN)_6_]_0.83_	K_0.05_Cu[Fe(CN)_6_]_0.67_	10 M NaClO_4_	0.65–1.35	26 (10C)	No decay (1000)	[140]
K_0.01_Mn[Cr(CN)_6_]_0.72_	Na_2_MnFe(CN)_6_	17 M NaClO_4_	0.5–2.6	37 (200)	84 (100)	[143]
Na_0.04_Mn[Cr(CN)_6_]_0.70_	MnHCFe	17 M NaFSI	− 0.97– − 0.5	62 (15)	–	[144]
Na_1.24_Mn[Mn(CN)_6_]_0.81_	CuHCF	1 M NaClO_4_	− 0.9– − 0.5	67 (60)	98.5 (700)	[141]
NaxMn[Mn(CN)_6_]	Pt	10 M NaClO_4_	− 1.2– − 0.6	85 (5000)	97 (3000)	[142]
Na_3_MnTi(PO_4_)_3_	Na_3_MnTi(PO_4_)_3_	1 M Na_2_SO_4_	0.4–1.8	57.9 (29.3)	98 (100)	[43]
Na_2_Ti_3/2_Mn_1/2_(PO_4_)_3_	NiHCF	6 M NaClO_4_	− 1.0–0	88.6 (0.5C)	90 (1000)	[146]

## Summary and Perspectives

Aqueous sodium-ion batteries are promising candidates for large-scale energy storage systems because of abundant sodium resources, low cost, high safety, convenient manufacture and eco-friendliness. The electrode materials and aqueous electrolytes affect the electrochemical performance of aqueous batteries. In this review, the recent development of Mn-based electrode materials for aqueous SIBs, including oxides, Prussian blue analogues and polyanion compounds, are overviewed. The electrochemical performance and improvement methods of Mn-based electrode materials are highlighted. The reported progress of Mn-based electrode materials is focused on cathode materials, oxides cathode materials in particular. However, the Mn-based Prussian blue analogues and Mn-based polyanion compounds also show high specific capacities and good cycling performance. It is believed that the Mn-based electrode materials are promising materials for aqueous SIBs. Therefore, the Mn-based electrode materials are worthy of further investigation. The following is some perspectives of Mn-based electrode materials in aqueous SIBs.

### Mn-Based Electrode Materials

Mn-based electrode materials include oxides, Prussian blue analogues and polyanion compounds, and each type of material has its distinctive advantages and disadvantages. Mn-based oxides materials have high theoretical capacity, however, most of them also suffer from phase transitions, leading to structural degradation and capacity decay during Na ions intercalation/deintercalation. Also, Mn dissolution and water protons co-insertion are some issues in aqueous electrolytes. Therefore, some improvement methods have been developed to alleviate these problems, including electrolyte optimization, morphology optimization, element doping or substitution, and carbon modification. For electrode materials, the synthesis of materials is very important, which determines the morphology and carbon coating quality of materials. Continued efforts should be devoted to optimizing material morphology (nanostructured materials in particular) combining with carbon modification in order to improve electrochemical performance of Mn-based oxides. In addition, element doping or substitution is also a powerful approach for improving the electrochemical performance. The effect of different dopants or element substitutions should be systematically investigated, which will provide a guide for designing advanced Mn-based materials with excellent electrochemical performance. For Mn-based Prussian Blue analogues, they have wide channels allowing rapid insertion/extraction of Na ions; however, they also suffer from poor cycling stability. Prussian blue analogues usually contain [Fe(CN)_6_]^4−^ vacancies, which affect the electrochemical performance. Reducing the number of [Fe(CN)_6_]^4−^ vacancies can improve its cycling stability [125]. However, the existence of unconventional Mn vacancies on the surface of Mn-based Prussian blue analogues is helpful to improve its long-term cycling stability [147]. Furthermore, carbon modification is also an attractive strategy to improve electrochemical performance of Mn-based Prussian blue analogues. For Mn-based polyanion compounds, their inferior electronic conductivity leads to low electrochemical performance. Surface-coating should be an effective way to enhance the electrochemical performance of Mn-based polyanion compounds [137]. Much attention should be devoted to these improvement strategies, which are related to material synthesis. The composition, crystal structure, morphology, element doping or substitution, and carbon modification are able to be controlled and optimized during synthesis process of materials. Therefore, the material synthesis is very important, where some improvement strategies could be combined together to enhance greatly the electrochemical performance.

### Aqueous Electrolytes

Aqueous electrolytes affect the electrochemical performance of Mn-based electrode materials. Optimizing the aqueous electrolytes is conducive to increase specific capacity and improve cycling stability of Mn-based electrode materials. Some optimization methods, such as optimizing electrolyte salt concentration, choosing suitable electrolyte additive and optimizing solvent, have been developed. The high concentration electrolytes or “water-in-salt” electrolytes can widen the stable electrochemical window, suppress Mn dissolution and some side reactions between electrode and water, and significantly improve the cycling stability of Mn-based electrodes. Therefore, developing innovative high concentration electrolytes or “water-in-salt” electrolytes is a promising way to obtain high electrochemical performance of aqueous batteries. In addition, the use of electrolyte additives, for example SDS [22, 95], in low concentration electrolytes could expand the electrochemical window, inhibit the water decomposition, suppressed the Mn dissolution, and improve the rate capability and cycling stability of Mn-based electrodes. Moreover, the addition of Zn, Mn and Li cations in low concentration electrolytes can also improve effectively the electrochemical performance of Mn-based electrode materials [93, 94]. Thus, the use of electrolyte additives is a very attractive improvement strategy. The effect of different electrolyte additives should be systematically studied, and the joint use of multiple additives, for example SDS and metal cations, should also be considered, which may have immense effect on enhancing electrochemical performance. Furthermore, hybrid electrolyte and eutectic electrolyte have been also proven effective to improve the electrochemical performance of Mn-based electrode materials, and they are worthy of further investigation.

For practical application in aqueous SIBs, it is expected that Mn-based electrode materials can exhibit high special capacity, superior rate capability and excellent cycling performance. Thus, high-performance Mn-based electrode materials need to be designed and developed. However, for aqueous SIBs, aqueous electrolytes have an important impact on the electrochemical performance of batteries. Optimizing aqueous electrolytes is a very effective approach for improving the electrochemical performance of Mn-based electrode materials. In especial, the expanded electrochemical stability window of aqueous electrolytes can allow the full and reversible use of sodium storage sites in Mn-based electrode materials, which will greatly enhance the electrochemical performance. Therefore, more efforts should be made to optimize aqueous electrolytes in addition to developing high-performance Mn-based electrode materials.
